# Surgical Sabermetrics: A Scoping Review of Technology-enhanced Assessment of Nontechnical Skills in the Operating Room

**DOI:** 10.1097/SLA.0000000000006211

**Published:** 2024-01-23

**Authors:** Emma E. Howie, Olivia Ambler, Eilidh G.M. Gunn, Roger D. Dias, Stephen J. Wigmore, Richard J.E. Skipworth, Steven J. Yule

**Affiliations:** * Clinical Surgery, University of Edinburgh & Royal Infirmary of Edinburgh, Edinburgh, Scotland; † Edinburgh Surgical Sabermetrics Group, University of Edinburgh, Edinburgh, Scotland; ‡ Human Factors and Cognitive Engineering Lab, STRATUS Centre for Medical Simulation, Brigham & Women’s Hospital, Boston, MA; § Department of Emergency Medicine, Harvard Medical School, Boston, MA

**Keywords:** cognitive load, nontechnical skills, objective measures, performance, surgical sabermetrics

## Abstract

**Objective:**

To evaluate the current evidence for surgical sabermetrics: digital methods of assessing surgical nontechnical skills and investigate the implications for enhancing surgical performance.

**Background:**

Surgeons need high-quality, objective, and timely feedback to optimize performance and patient safety. Digital tools to assess nontechnical skills have the potential to reduce human bias and aid scalability. However, we do not fully understand which of the myriad of digital metrics of performance assessment have efficacy for surgeons.

**Methods:**

A systematic review was conducted by searching PubMed, EMBASE, CINAHL, and PSYCINFO databases following PRISMA-ScR guidelines. MeSH terms and keywords included “Assessment,” “Surgeons,” and “Technology”. Eligible studies included a digital assessment of nontechnical skills for surgeons, residents, and/or medical students within an operative context.

**Results:**

From 19,229 articles screened, 81 articles met the inclusion criteria. The studies varied in surgical specialties, settings, and outcome measurements. A total of 122 distinct objective, digital metrics were utilized. Studies digitally measured at least 1 category of surgical nontechnical skill using a single (n=54) or multiple objective measures (n=27). The majority of studies utilized simulation (n=48) over live operative settings (n=32). Surgical Sabermetrics has been demonstrated to be beneficial in measuring cognitive load (n=57), situation awareness (n=24), communication (n=3), teamwork (n=13), and leadership (n=2). No studies measured intraoperative decision-making.

**Conclusions:**

The literature detailing the intersection between surgical data science and operative nontechnical skills is diverse and growing rapidly. Surgical Sabermetrics may provide a promising modifiable technique to achieve desirable outcomes for both the surgeon and the patient. This study identifies a diverse array of measurements possible with sensor devices and highlights research gaps, including the need for objective assessment of decision-making. Future studies may advance the integration of physiological sensors to provide a holistic assessment of surgical performance.

Surgeons, like elite athletes, are in constant pursuit of performance enhancement to improve outcomes for patients and the systems they work in. However, the current climate of increased service demands and pressure, significant waiting list burden, reduced training opportunities, increasing health care attrition rates, and the impact of incivility means that surgeons often do not receive the necessary feedback to optimize performance. The importance of nontechnical skills in enhancing surgical performance and patient safety is now well-established and tools for individual and team assessments have sufficient validity evidence to be identified as gold standards.^1,2^ The challenge is that human assessments of surgical performance are limited by bias, time, cost, and scalability. To meet the growing demand for objective evaluation of operative nontechnical skills, researchers have trialed automated, digital metrics which promise true, accurate, real-time feedback.^3–5^ Termed surgical sabermetrics, the practice of digital assessments for feedback and improvement in surgery is influenced by performance monitoring in elite sports, where multimodal data are integral to training, deliberate practice, and winning championships.^6^ These assessments integrate audio-visual capture of the operating room (OR) with sensor-based digital assessment of the surgeon and operative team, and clinical data to provide advanced holistic analytics of surgical performance.^6^

The focus of this review is nontechnical surgical performance, with the nontechnical skills investigated as those described by the Non-Technical Skills for Surgeons (NOTSS) taxonomy: Situation Awareness (SA), Decision-Making, Communication, Teamwork, and Leadership.^2^ Regardless of competence and expertise, surgeons are subject to the cognitive benefits and limitations of the human brain.^7^ In fact, nontechnical skills are defined as “cognitive, social and personal resource skills that complement technical skills and contribute to safe and efficient task performance.”^8^ It is taking this definition into consideration that this review introduces Cognitive Load (CogL) as an additional nontechnical construct. CogL is a concept that underpins expert performance in surgery. Defined as the amount of finite working memory resources an individual must allocate to meet the cognitive demands of a task,^9–11^ CogL is a multidimensional concept^12,13^ and excessively high levels may have negative consequences for individual learning, team performance, and patient safety.^6,14^ Despite being different concepts, there is overlap in the literature between CogL and stress,^15^ the feeling of strain or threat,^16,17^ and the same methods have been used to measure these different cognitive states.^18,19^ Increased stress and CogL have both been shown to have a negative effect on surgeons’ nontechnical skills.^20^

Objective performance metrics can be broadly classified as either physiological (eg, cardiovascular, respiratory, dermatological, neurological, optical, or energy expenditure) or nonphysiological (eg, movement and acoustic analysis). Changes in CogL can be detected in an individual’s physiology due to activation of the autonomic nervous system (ANS).^21^ Sensor-based objective metrics can measure the ANS directly and are used as surrogate measures of CogL or proxy assessments of nontechnical skills.^7,22^ Examples of physiological indicators include electrodermal activity (EDA), electroencephalography (EEG), and heart rate variability (HRV).^23,24^

Previous reports on nontechnical skills in surgery have been conducted in the context of human assessments of behavior,^25^ surgical education,^25^ human factors considerations,^26^ and cognitive load measurement.^26^ The rise of technology presents a remarkable opportunity to explore the application of measuring nontechnical skills in a surgical context, yet the extent of its utilization remains unclear. The aim of this scoping review is to evaluate the current technological advances in measuring surgeons’ nontechnical skills using objective metrics. The present study also evaluates the various biomarkers used to measure nontechnical skills, and the specific surgical contexts in which surgical sabermetrics tools have been implemented. Furthermore, we intend to discuss the interventions included in these studies aimed at improving surgical performance.

## METHODS

A scoping review was conducted in August 2022, following the Preferred Reporting Items for Systematic reviews and Meta-Analyses extension for Scoping Reviews (PRISMA-ScR) checklist as guidance^27^ (Supplemental Digital Content, http://links.lww.com/SLA/E999). The Population Intervention Context framework^28^ was utilized to develop the search strategy with the expertise of a professional medical librarian. This review is registered by submission to *BMJ Open.* ^29^ Systematic searches of PubMed, OvidMedline, Embase, PsycINFO, IEEE Xplore, Web of Science, and ACM digital library databases were performed from inception to August 2022, using Covidence Software (Veritas Health Innovation) to collate manuscripts. MeSH terms and keywords included, but were not limited to “Assessment,” “Surgeons,” and “Technology,” in addition to terms relating to nontechnical skills. A full example of search terms including MeSH terms and keywords is included in Supplemental Digital Content (Appendix A, Supplemental Digital Content 1, http://links.lww.com/SLA/E999). Reference lists were not cross-searched.

### Selection Criteria and Screening Process

**FIGURE 1:**
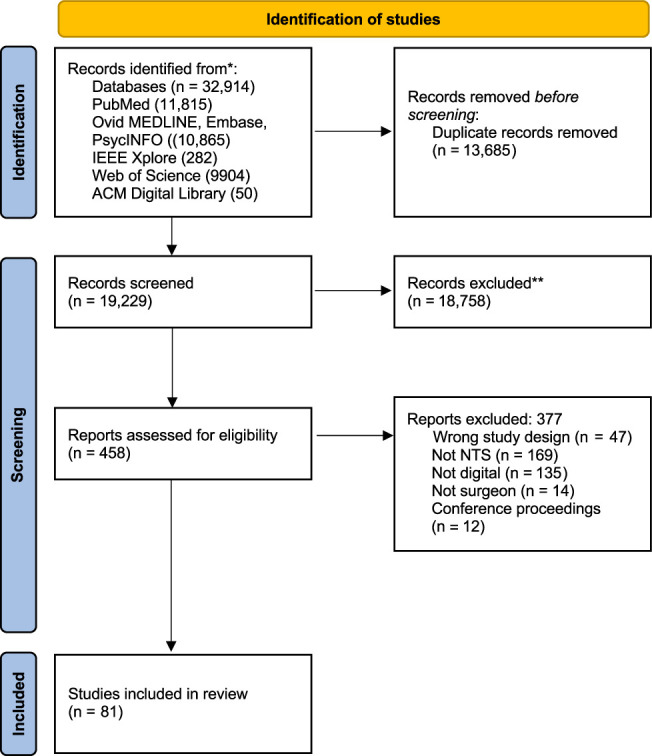
PRISMA diagram. NTS indicates nontechnical skills.

We aimed to include all original research studies published since 2010 to capture technology advances over the past decade. Included studies involved the digital measurements of nontechnical skills, following the NOTSS taxonomy and including CogL, in a surgical context. The target population included surgeons from all surgical specialties and training levels (trainees, residents, and fellows), in addition to attending (consultant) surgeons. Studies utilizing real-life and simulated surgical environments were included. We excluded studies where full text was not available, that were not written in English, that did not measure nontechnical skills, that targeted the incorrect population, or did not report surgeon data, along with the following article types: review articles, conference abstracts, and letters to the editor. Following explicit inclusion and exclusion criteria, abstracts were screened by 3 reviewers (E.E.H., O.A., and E.G.M.G.) to identify articles that would later be assessed by a full-text review. The same authors conducted a full-text review of included papers against the inclusion/exclusion criteria. Conflicts that arose either during the title/abstract screen, or full-text review, were resolved by a fourth author (S.J.Y.). The PRISMA flow diagram detailing the number of articles screened and included is shown in Figure 1.

### Data Extraction

Data extraction was conducted by 3 authors (E.E.H./O.A./E.G.M.G.) after calibration with a fourth author (S.J.Y.) on extraction criteria. During this process, the fourth author was consulted on study ambiguities or study data that did not clearly fit the predetermined extraction criteria. Data extraction was cross-checked by 2 authors (E.E.H./O.A.). Detailed information was extracted from each study under several categories including study design, participant cohort, and nontechnical skill assessment. Extracted data were captured using a dedicated spreadsheet designed for this review.

### Data Synthesis and Quality Assessment

Data were analyzed using quantitative and descriptive statistics. A qualitative narrative synthesis was conducted to identify themes demonstrating how sabermetrics has been applied in the surgical context, presented using visualizations and flow diagrams. These include tables focusing on the scope and aims of included studies; quality of methods; participant analysis; contextualization; specific nontechnical skills assessed; metrics used; and study conclusions. Quality assessment of individual papers was undertaken using the Quality Assessment Tool for Diverse Designs (QATSDD).^30^ The QATSDD score was calculated by 2 authors (E.E.H./O.A.) based on the application of standardized and validated criteria.

## RESULTS

The results of the database searches are outlined in a PRISMA diagram (Fig. 1). The overall summary of the 81 included studies is available in Supplemental Table 1, Supplemental Digital Content 1, http://links.lww.com/SLA/E999. The details of the included studies (eg, participants, study design, aims, and outcomes) were significantly heterogeneous. The proportionate inter-rater reliability for full-text review was 0.94.

### Participants

There were a total of 1335 unique study participants represented across studies (mean, n=17, range, 1–121). Attending surgeons (n=152/1335, 11%), residents (n=453/1335, 34%), medical students (n=236/1335, 18%), unspecified surgical roles (n=473/1335, 35%), and nonmedical personnel (n=21/1335, 1.5%). Two studies did not declare the sample size.^24,31^

Eighteen studies (n=18/81, 22%) did not specify the role or level of the surgeon. Where the surgeon role was specified, studies most commonly measured the sabermetrics of individual trainee surgeons (n=17/81, 21%). There were 9 studies (n=9/81, 11%) assessing the sabermetrics of individual attending surgeons.

Twelve studies (n=12/81, 15%) assessed surgeons as part of the wider operating team, including anesthesiologists and nursing staff. Eleven studies examined attending surgeons and residents operating together (n=12/81, 15%). Three studies (n=3/81, 4%) looked at medical students with a surgeon (n=2) or in a team (n=1). Studies that recruited solely medical students (n=7/81, 12%) or nonmedical personnel (n=2/81, 2%) were included only if they incorporated a surgical task, such as laparoscopic simulation.

### Study Setting and Surgical Modality

Of the 81 studies, the majority were conducted in simulation (n=48/81, 63%), compared with real surgery (n=32/81, 40%) (Supplemental Table 1, Supplemental Digital Content 1, http://links.lww.com/SLA/E999). A single study^24^ compared simulation and real surgery. The majority of surgical modalities were minimally invasive (n=52/81, 64%) or open surgery (n=18/81, n=21%), with the remaining studies incorporating a combination of surgery modalities or generic surgical skills. Studies addressing minimally invasive surgical modalities covered laparoscopic (n=32/52, 62%), robotic (n=14/52, 27%), endoscopic surgery (n=3/52, 6%), or a combination (n=3/53, 6%).

### Study Design, Settings, and Characteristics

The number of included studies increased each year from 2010 to 2022, demonstrating the rise in popularity of surgical sabermetric monitoring in surgery. Included studies covered a breadth of surgical specialties including gynecology (n=2/81, 2.5%), orthopedics (n=3/81, 4%), general surgery (n=19/81, 23%), cardiothoracic surgery (n=10/81, 12%), and urology (n=15/81, 19%). Twenty-two (n=22/81, 27%) studies did not specify a surgical specialty, and 5 (n=5/81, 6%) involved multiple specialties in the same study. The majority of studies arose from the United States (n=28), the United Kingdom (n=9), China (n=8), Canada (n=7), and Germany (n=5). Forty-one studies combined objective and subjective measures. Only 3 randomized controlled trials were identified in the reviewed literature, one of which was a multicenter trial. Of the included studies, only 1 additional study was multicentric. Studies were commonly framed as pilot (n=21) or feasibility (n=4) and were often observational (n=26) or interventional (n=13) in nature. Only 1 study compared simulation and live surgery.

### Nontechnical Skills Assessed

Within the included studies, 52% (n=42/81) measured at least 1 nontechnical skill. Individual nontechnical skills measured included SA (n=24/42, 57%), teamwork (n=13/42, 31%), communication (n=3/42, 7%), and leadership (n=2/42, 5%). The only NOTSS category not included in any study was decision-making. Thirty studies (37%) investigated 2 or more nontechnical skills concurrently. The majority of studies also measured either CogL (n=57/81, 70%) or stress (n=19/81, 23%) with 3 studies examining both concepts together. Supplemental Table 2, Supplemental Digital Content 1, http://links.lww.com/SLA/E999 maps sabermetric concepts and biomarkers against NOTSS domains.

### Objective Metrics

**FIGURE 2:**
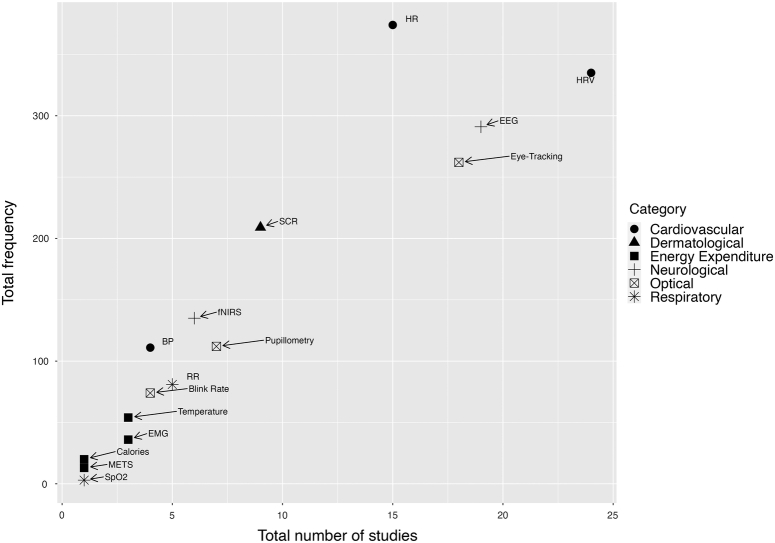
Frequency of metrics in studies versus overall. Key: Cardiovascular (N=817): HRV (n=333), HR (n=373), and BP (n=111); dermatological (N=204): EDA (n=204); energy expenditure (N=118): calories expended (n=20), METs (n=8), EMG (n=36), Temperature (n=54); neurological (N=391): EEG (n=256), fNIRs (n=135); optical (N=432): eye-tracking (n=262), pupillometry (n=96), blink rate (n=74); respiratory (N=84): RR (n=81), SpO_2_ (n=3). BP indicates blood pressure; EMG, electromyography; METs, metabolic equivalents; RR, respiratory rate.

**FIGURE 3:**
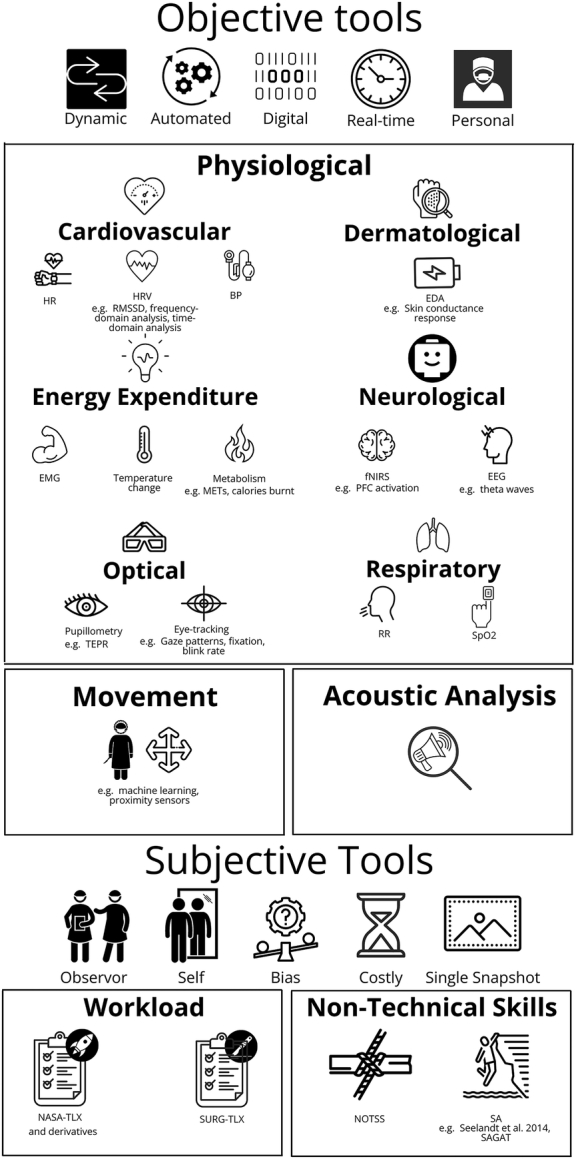
Summary of objective and subjective assessment tools.

In this comprehensive review of 81 studies, a diverse array of measurements were implemented, with a total of 122 distinct objective, digital metrics utilized across 16 separate categories. The automated, objective measurements of nontechnical skills (NOTSS) in this study were categorized into 2 categories: physiological and nonphysiological metrics (Figs. 2, 3). Surgeon physiological measurements (n=115) using noninvasive sensors were the preferred measurement of choice, but technology such as acoustic analysis (n=3)^32–34^ and movement (n=4)^33,35–37^ were also employed. Cardiovascular measurements were the most common physiological metrics employed (n=46), which include HRV, heart rate (HR), and blood pressure. HRV was the most frequently used measurement across all included studies (n=25). The next most common categories were neurological (n=24) with EEG (n=18) and functional near-infrared spectroscopy (fNIRS) (n=6), then optical (n=21). Figure 2 compares the number of studies using a physiological metric (*x*-axis) versus the frequency a metric was used by a study participant (*y*-axis), where the sample size was known. This figure shows that HRV is also the most common metric in terms of participants (n=333), but HR (n=373), eye-tracking (n=262), and EEG (n=256) have also been deployed frequently.

Twenty-seven studies (34%) combined 2 or more objective metrics. HRV was the most frequently selected metric to use in combination (n=12). Curiously, fNIRS was always used with another metric, and typically with a subjective measurement (n=5). The less frequently utilized metrics also tended to be used alongside others. For example, electromyography (n=3) was only used in combination with other metrics. Considering 39% (n=31) of studies involved live operating, there was a disproportionate use of metrics in live surgery settings compared to simulation. For example, eye-metrics were only used in 4 (5%) of the live setting compared to 21 studies overall. Similarly, EEG was only used 5 times in live surgery versus 18 overall. From the simulation studies, there is a clear need for these metrics, which must now be translated into clinical practice.

### Subjective Metrics

Forty-one studies combined objective and subjective, nondigital measurements. Three studies concurrently utilized human raters and the NOTSS taxonomy alongside a digital measurement. Thirty-two studies utilized subjective CogL measurements with tools including the Surgical Task Load Index (SURG-TLX) (n=8)^38^ and NASA Task Load Index (NASA-TLX) (n=23).^31,38^ One study used a behavior marking system by Seelandt and colleagues to assess distractions.^32,39^ Three studies utilized the Observational Teamwork Assessment for Surgery (OTAS).^31,40–42^ Eight studies used the subjective State-Trait Anxiety Inventory (STAI) to assess perceived stress. Objective measures such as EDA were significantly related to these subjective, self-rated measures.^43,44^

### Outcomes

Improving outcomes and reducing error are common aims of sabermetrics studies. Outcomes measured alongside surgical applications in included studies can be broadly classified as those that evaluate (i) surgical performance and patient safety,^44^ or (ii) surgeon well-being^45^:

- Surgical performance and patient safety: One specific use of sabermetrics for patient safety included using HRV to evaluate the CogL associated with an error as part of a root-cause analysis^46^ Roberts et al^47^ described a link between technical skills, CogL, and error, showing that surgeons with higher CogL are less likely to accept help or change tactics, often sticking with ineffective strategies which can cause harm.^48–50^ The use of sabermetrics may reassure patients that surgeons are working in optimal conditions and that a new technology or technique does not place unnecessary additional CogL on the operating surgeon that may negatively affect performance.
- Surgeon well-being: Physiological measurements can also be used to support well-being. HRV measurements have been used to identify daily stressors and periods of increased CogL associated with physiological strain.^51^ Issues such as fatigue, burnout, anxiety, and depression have been associated with EEG and EDA changes.^43,52,53^ Poor performance or being involved in an error contributing to patient harm can cause moral injury,^54^ and continual monitoring of performance through sabermetrics could reduce this risk.

### Practical Application of Sabermetrics Implementation

A breakdown of the practical application of sabermetrics implementation in included studies is demonstrated in Table 1. The practical applications can be categorized into 13 overriding categories. The majority of studies used sabermetrics to measure task demands (n=24), for example, a surgeon adjusting to the demands of the surgical task and environment or increasing case difficulty. Sabermetrics were also used to examine performance modulators such as noise,^32,68,69^ or the effect of an intervention, such as intraoperative breaks or a training technique.^41,53,55,69,70^ Many studies underwent user testing in technological innovation of sabermetrics, including assessing the feasibility, reliability, or validity of methods of data collection or analysis for NOTSS and performance assessment using automated technology such as sensors or computer vision.^7,24,36,37,49,67,71–75^ A further application was to compare the effects of different surgical modalities, for example, laparoscopic versus robotic surgery.^60,76–80^

**TABLE 1:** Outline of the Practical Applications of Sabermetrics Implementation in Included Studies

Primary purpose	Example	Metric used
Examine effect of external factors on performance	Engelmann et al^55^ Gao et al^56^ Yu et al^57^	HRV Eye-metrics EEG, HR
Measure task demands	Jiang et al^58^ Zhang et al^59^	Eye-metrics
Compare different surgical modalities	Stevens et al^60^	EEG
Quantify expertise	Gunawardena et al^61^ Wilson et al^38^	Eye-metrics EDA
Quantify stress	Jukes et al^62^ Pimentel et al^14^	BP, HR HRV
Quantify cognitive load	Rieger et al^35^	HR, Movement, RR, Temperature
Evaluate training efficacy	Feeley et al^63^	Calories burned, HR
Evaluate a nontraining intervention, eg, breaks	Engelmann et al^55^	HRV
Individual performance assessment	Zheng et al^64^	Eye-metrics
Team performance assessment	He et al^65^	Eye-metrics
Analyze patient safety incidents	Zenati et al^7^ Roberts et al^47^	HRV Eye-metrics
Support provider well-being	Kahol et al^52^	EEG
User testing in technological innovation (eg, feasibility/sensitivity, innovation)	Arora et al^66^ Shafiei et al^67^	HRV EEG

BP indicates Blood Pressure; METs, metabolic equivalents; RR, respiratory rate.

### Quality of Studies

The quality of studies was highly variable according to the Quality Assessment Tool Studies with Diverse Designs (QATSDD). QATSDD scores ranged from 25% to 81% (median=57%, interquartile range=17%, where Q1=48%, Q3=64%) with a higher score indicating higher quality. The median quality score for studies that utilized subjective and objective measures was 57% (IQR: 50%–64%). Less than half of the included studies (n=37) examined the reliability and validity of the measurements selected. In addition, the median score given for the assessment of reliability and validity was 0 (out of 3).

## DISCUSSION

### Overview

In this scoping review, we aimed to present a comprehensive picture of the available research on the use of automated, real-time, objective measurements for the assessment of surgeons’ operative nontechnical skills, characterized as surgical sabermetrics.^80–82^ The literature demonstrates that surgical sabermetrics has been assessed in both real operative and simulated surgical settings, with measurements from a range of physiological sensors, combined with observations, video, and surveys to add context. The included studies highlight the importance of CogL in the assessment of surgical performance, with clear evidence of its influence on surgeons’ behavioral and cognitive skills across applications. SA was the most commonly measured nontechnical skill and its measurement was commonly entwined with CogL.^81–83^ There was substantial heterogeneity among the 81 studies that met the inclusion criteria, especially around study designs and measured outcomes. The current sabermetric monitoring and interventions that have been applied in surgery lack standardization; however, the included studies provide evidence of feasibility and direction on research gaps in this rapidly evolving field.

### Metrics

Cardiovascular metrics were the most utilized objective measurement and were likely selected due to their evidence base and relative ease of measurement via consumer devices.^84^ Studies utilizing eye-tracking metrics opted for wearable glasses-style or head-mounted devices. Eye-tracking bar-type devices are also available, which have been used in nonsurgical settings but not within surgery to our knowledge.^85^ It is possible to attach these devices to a laparoscopic monitor, but this would not be suitable for robotic or open surgery. In addition, neurological measurements such as EEG and fNIRS often require participants to wear a full “cap,” or smaller headband-like EEG device. Specifically, fNIRS gives an indication of energy use for cognitive tasks by measuring blood oxygenation levels in areas of the brain, typically the prefrontal cortex, but has only been used thus far in combination with cardiovascular metrics.^18,86^ Dermatological measures such as EDA are markers of ANS activity measured through subtle changes to electrical activity within our skin as detected by electrodes.^87^ Energy expenditure methods involve using temperature devices such as thermal cameras, or estimated calorie expenditure through HR devices. Assessment of movement within the OR involved sensors or machine-learning techniques with video recordings, utilizing commercial recording equipment and open-source algorithms demonstrating that low fidelity methods of assessment are possible.^37^

### Cognitive Load

Real-time measurement, modulation, and optimization of CogL can enhance surgical performance. Measured through ANS changes or via the metabolic demands of carrying out a task as evidenced by metrics of the included studies, CogL is a proxy for NOTSS measurements and has a variety of wider implications and applications. Measuring CogL indicates the current cognitive effort being expended and suggests the residual capacity available.^49^ Increasing CogL can impair individual and team technical and nontechnical performance, putting patient safety at risk. None of the reviewed studies identified specific absolute levels of “overload.” Only 1 study attempted to quantify optimal levels of CogL, utilizing the index of pupillary activity, utilizing eye-tracking with an index of cognitive activity.^47^

Intraoperative stressors and prolonged attention contribute to higher levels of stress and CogL.^32,56,88–91^ Conversely, performing a task at a lower CogL increases the chance of proficiency, and allows for the residual capacity and mental effort to be redistributed to deal with other tasks or saved for periods of increased task demands.^80^ Increased CogL has been linked to musculoskeletal pain in nursing staff but its effects on well-being have not yet been objectively investigated in the surgical team.^92–95^ There is an association between increased CogL and muscle activity, leading to muscle fatigue and subsequently to pain and discomfort.^96,97^ Monitoring and optimizing CogL may aid in the reduction of pain and discomfort, essential as surgeons are at risk of work-related musculoskeletal injury which can impact individual well-being and the overall workforce.^98,99^ Further applications of CogL are detailed within the outcomes and applications of the sabermetrics sections of this review.

### Nontechnical Skills

The NOTSS taxonomy^51,56,88^ was used to classify objective metrics according to the main categories of surgical nontechnical skill in Supplemental Table 2, Supplemental Digital Content 1, http://links.lww.com/SLA/E999. Neurological measurements, such as EEG and fNIRS, were used to provide a direct indication of SA through measuring attention and engagement.^52,89,91^ Objective measurements support the hypothesis that acute stress can impair SA and surgical performance through the mechanism of increasing CogL.^24,56,90,100,101^ Increased surgeon reactivity to intraoperative stressors, demonstrated through CogL measurement, was found to indicate a loss of SA.^102^ High CogL decreases SA by affecting attention, increasing reaction time, and negatively impacting recognition skills.^24,103,104^ Measuring overall team CogL through individual team member measurements can show psychophysiological mirroring and dynamic CogL changes occurring during unexpected or expected, task-specific events and provide evidence of effective teamwork.^7,24,64,105,106^ Social proximity is a predictor of behavior as it influences the opportunities available for effective teamwork and communication through nonverbal interactions and is suggestive of a shared mental model.^33,37^ Studies have shown that computer vision techniques and proximity sensors for movement analysis can demonstrate an association between movement and teams with “poor” and “good” SA in the OR. Proximity sensors demonstrated the movement and closeness of the team around each other with “good SA” associated with restricted movements. The time a team member spent close to the primary operating surgeon was a predictor of their NOTSS score.^33,37^ Although no studies assessed decision-making directly, it is a complex and dynamic cognitive process that is intertwined with other nontechnical skills.^2,107^ The effect of SA, stress, and increased CogL may cause detrimental effects on the human memory system, resulting in decision fatigue, tunnel-vision, and premature decision-making.^2,108,109^

### Applications of Surgical Sabermetrics

Sabermetrics methods are beneficial as they reduce reliance on human observers, which increases the speed, volume, objectivity, and value of assessments. Digital measurements also remove the risk of biased judgments often associated with subjective assessments. Automated assessments also have the benefit of discreetly measuring performance in real time for analysis and interpretation at a later date without interrupting the target procedure or surgical workflow.

A number of applications emerged from the literature, covering topics as diverse as surgical training, systems design, modality, and feedback. These have implications for the wider surgical team, including trainees and patients. Training applications include the optimization of CogL for surgical residents and assessment of the efficacy of training interventions. For example, Wu et al found that using cognitive and behavioral metrics through EEG and eye-metrics, with machine-learning can predict training outcomes with a 72.5% accuracy.^110^ EEG features can also detect high CogL and altered performance during training.^44,57,110–113^ Optimizing CogL in trainees can improve their capacity for learning and progression.^114–116^ In one example, Maimon et al (2022) used CogL levels via EEG in simulation to assess if a trainee was “ready” to operate on a live patient.^117^ This application offers immense utility in allowing for a tangible demonstration of surgical skill progression, as training practices increasingly move toward the use of simulation to supplement training.^118^

Sabermetric measures have also explored the role of the trainers’ presence on trainee performance, and provide indications of trainer-trainee trust.^63,119^ Objective measures have the potential to contribute to formative and summative assessment, especially within a personalized education lens, for example, the longitudinal use of metrics to measure changes compared to previous.^112,116,120^

There have also been many nontraining applications, including the assessment of performance modulators’ use of technology, the role of time pressure, and the impact of rest breaks on performance^4,55,68,100,121,122^ Sabermetrics has been used to compare the CogL exerted by different modalities, including robotic versus laparoscopic surgery, live surgery versus simulation, and 2D versus 3D operative views.^60,76–80^ Measuring task demands was the most prevalent application. Surgical task characteristics can impose a high level of demand, driven by factors such as case difficulty, the need for precision, multitasking, and the use of adjuncts such as virtual reality.^58,103,123^ Objective measures can quantify the effect of these demands and determine if a new surgical tool, technique, or modality results in a substantial increase in CogL. Although there are currently no set criteria for cognitive overload in surgery, sabermetrics can be used to monitor CogL and optimize success by identifying contexts and trends that negatively impact operative performance.

### Concurrent Assessment of Nontechnical Skills Using Objective and Subjective Metrics

The current gold standard tools for assessing nontechnical skills and cognitive load in surgery are subjective and observational measures. The use of these validated, nondigital tools is widespread within surgery. Dominant examples include ratings provided by a trained observer using the NOTSS tool, and self-reported questionnaires in the form of the NASA-TLX. Although the aim of the present scoping review was to evaluate technological advances in measuring surgeons’ nontechnical skills using objective metrics, we anticipated that many included studies would also incorporate subjective and observational measurements. However, the majority of studies retrieved did not concurrently use subjective and observational methods alongside digital tools. When utilized, these measures were used for a variety of reasons. For example, Dias et al^37^ used human NOTSS assessments to assign operative teams to “low” and “high” SA, and then investigated digital movement metrics, comparing these groups, finding that teams with higher rated SA had lower entropy and therefore less movement during the surgical time out. Cha et al^33^ also gathered human NOTSS assessments for the purpose of correlating nontechnical skills scores with the speech and proximity metrics of participants. A number of human observation tools, including OTAS, Situation Awareness Global Assessment Technique (SAGAT), and NOTSS have also been used as outcome measures, to investigate the impact of operative stress on performance and teamwork.^31,42,69,104,124^ Workload measures, such as NASA-TLX and the surgical variant called SURG-TLX, were used to provide essential subjective data and support the evidence for objective measures. Subjective, self-report measures may provide context to objective metrics, and enhance our understanding of surgeons’ personal interpretation of operative performance. It is crucial therefore to combine objective and subjective measures to provide a complete picture of operative events.^125^

### Digital Measurement of Technical Skills

Good technical surgical skills are reliant on competence in both cognitive and psychomotor skills.^52^ Although the focus of this review was on nontechnical skills, some studies concurrently investigated technical skills and overall performance, through standard, human-rater–dependent measures such as Objective Structured Assessment of Technical Skill (OSATS) or the Generic Error Rating Tool (GERT).^23,69^ According to the inclusion criteria, studies had to measure some aspects of nontechnical skills (SA, decision-making, communication, and leadership) to be included, so studies that utilized sensors or digital technology to solely measure technical skills were excluded. However, the lines between technical and nontechnical surgical skills are blurred and it is possible that some of the assessments in the included studies are also reflective of the technical skills being performed. For example, economy of motion is generally accepted to be reflective of surgical technique, gained through experience, and described variously as “fluidity” and “efficiency” by surgeons. It features in the dominant surgery assessment tools, including OSATS, and is the basis of thousands of assessments in the surgical literature. However, effective economy of motion, fluidity, and efficiency are critically dependent on SA, a core nontechnical skill that is associated with higher-order thinking and the ability to gather, understand, and predict future states in dynamic situations such as operative surgery. In addition, a hallmark of expertise is the ability to leverage automaticity in surgical practice; what may appear effortless, smooth, and precise hand motion is a function of CogL management. Experienced surgeons are able to “free up” cognitive resources at the moment in what is labeled System 2 thinking.^125^ This scoping review reveals emerging evidence supporting the objective measurement of CogL in the operative setting. Notably, while cognitive load is a prerequisite for technical proficiency, its influence remains distinct and independent in nature. Several studies assessed the impact of stress and CogL on technical performance as an outcome. For example, Grantcharov et al^23^ found that high cognitive load and acute stress levels measured via HRV negatively impacted technical performance. Similarly, eye-tracking can be used to measure attention and other cognitive processes. Although none of the studies in the present review used eye-tracking to measure technical skills, gaze patterns vary between novice and expert surgeons,^126^ and therefore can be used as a marker of technical skill acquisition. However, such studies fall outside the scope of this review.

### Limitations

Scoping reviews are ideally suited to capturing the breadth of novel topics, but are limited in the depth of evidence they can cover.^86^ Several limitations must be considered when interpreting the results of this review and future utilization of surgical sabermetrics. While the theoretical basis and use of these metrics have been established in nonhealth care industries,^127^ the studies included in the present review lacked assessment of the practical usage of these sensors in live surgery. Simulated environments were more commonly utilized to evaluate the reliability and feasibility of devices.^36,44,72,128^ To protect patient safety, newly introduced technological devices must not interfere with the physical OR environment or cause interference with patient monitors or surgical equipment.^74,129^ Furthermore, it is essential to evaluate the wearability and comfort of sensors according to end users (surgeons) to deploy them successfully during surgery.^36^ Wearable technology can be intrusive and unsuitable for long procedures,^130^ and may interfere with concurrent equipment such as headlamps and loupes.^74,131^ In the present review, eye-tracking glasses were reported to not cause discomfort, negatively impact the visual field, or affect surgical performance.^132^ However, no assessment was made regarding likely interaction with loupes, headlamps, or eye protection, and most studies excluded participants who required vision correction, such as glasses, limiting the scalability of eye-tracking devices. Physiological measurements can be influenced by the OR environment, such as room temperature, and individual differences between surgeons.^127,133,134^ Physiological measures are also affected by the individual’s overall state; including physical activity levels, stress, sleep, digestion, caffeine intake, circadian rhythms, and medical conditions.^44,135^ These confounding factors were not well-controlled across studies. Furthermore, many studies only assessed male surgeons, or excluded females to control the potential effect of stress response during menstruation,^136^ which is not demonstrative of the actual surgical landscape.

The objective metrics identified in the present review are directly measured continuous variables, characterized as proxy measures of the performance variables of interest (eg, cognitive load, SA, attention, stress, communication, and leadership). Interpretation of the surgical context is required to derive meaning, and ground truth is required to determine the validity. For example, eye-tracking metrics such as gaze pattern and fixation rate provide an indication of the psychological variable “attention,” but the attention may be misplaced. Specific, narrow gaze patterns can be variously interpreted as (i) deliberate focus during a surgical task, with effortful deprioritization of distractors, or (ii) “tunnel-vision,” a potentially dangerous state of cognitive bias reflecting loss of global SA. Alternatively, gazing around the OR, and directing attention from the surgical field to the patient monitor or anesthesiologist may be characterized as maintaining good awareness of the overall situation in the OR, or a sign of distraction and lack of focus. An integrative review combining objective metrics and audio-visual recordings offers a comprehensive perspective by providing contextual information and addressing these variations in interpretation.

In addition, measurements such as EDA can indicate levels of task engagement but cannot distinguish whether the engagement was appropriate and task-relevant or inappropriate and at risk of degrading SA and subsequent performance.^88^ Acoustic analysis can measure communication metrics relating to noise, including speech,^33,34^ with Cha et al^33^ noting a correlation between NOTSS scores and pitch, suggesting a link between speech metrics and leadership and teamwork behaviors. However, acoustic analysis cannot assess the content of speech. Noise peaks are associated with an increase in conversation that may be positively attributed to increased communication between team members.^33^ However, that conversation may be task-irrelevant, impacting teamwork and increasing CogL.^32^ Despite these illustrated limitations in interpretation, metrics such as HRV and EDA may offer unique insights as adjuncts to current performance metrics.

### Recommendations for Future Research

The heterogeneity of the study designs, data analysis, and outcomes means that a meta-analysis would be premature at this stage. The emerging evidence base and rapid development of sensor technology also make it difficult to draw firm guidance on practice changes for surgeons or recommend specific metrics to capture during live surgery. In line with previous reviews in surgery and other safety-critical industries, we did not find a consensus to recommend a single, objective physiological measurement of CogL.^127,137^ However, the present study advances knowledge beyond prior reviews by synthesizing the literature regarding automated, digital, objective measures for the assessment of nontechnical skills in surgery.^18,26,138^ On the basis of this synthesis, we make several recommendations regarding the future research agenda in surgical sabermetrics:

1. The use of a concurrent, validated subjective tool such as NOTSS to provide further evidence of the validity of objective metrics.
2. The concurrent use of audio-visual recording technology to provide context to data interpretation, rather than relying on human observation. This will add a true richness to the data and aid further research into metrics with conflicting interpretations. For example, acoustic analysis can detect noise peaks as a sign of increased team communication, and combining audio-visual recording with acoustic analysis may provide insight into the content of this communication.
3. In addition, combining the metrics of CogL with other objective measures of nontechnical skills, such as acoustic analysis for communication, may aid in the assessment of communication content and its relevance during cognitively challenging tasks.
4. The current heterogeneity of study designs limits the ability to directly compare studies or make recommendations; therefore, further studies with consistent reporting, measurement, and interpretation of data are required. For example, although there are several studies utilizing HRV, it can be assessed using time or frequency domains and therefore not all HRV studies are directly comparable.
5. Studies should include information on device usability including comfort to assess the impact on the wearer while operating.
6. Investigation into the acceptable range for optimal CogL, as there is currently no established upper or lower limit.

The studies in this review have demonstrated the wide range of benefits of these measurements and demonstrated the applicability of sabermetrics within the OR.

## CONCLUSIONS

Surgical sabermetrics is a novel and innovative area focusing on optimizing surgical performance to benefit both surgical teams and patients. The reach of sabermetrics is evident through the 81 studies in this scoping review, with clear merit in enhancing surgical well-being, performance, patient safety, and training. Despite being unable to draw a firm conclusion on which metric is “best,” there are still clear clinical benefits. This review shows that objective assessment of CogL is an established area of interest for surgeons; however, more research is required to investigate the objective assessment of nontechnical skills. The importance of nontechnical skills in surgery is well-established; however, the measurement of CogL as an indicator of mental processes has yet to be integrated into surgical practice. Although it is a concept rather than a skill in itself, the ability to effectively manage and optimize CogL is a skill that can lead to enhanced surgical performance and well-being.^139^

Further areas of research in this evolving field should investigate objective NOTSS assessment in the real OR and assess factors affecting uptake and usability in the OR with real usability data. The integration of current subjective assessments such as NOTSS, with objective measures of performance, audio-visual operative recordings, postoperative debriefing, and cognitive task analyses will provide rich performance assessment to improve surgical care.^107,140^ The studies included in this review demonstrate the importance of assessing cognitive load,^139^ and the feasibility of measuring several nontechnical skills indirectly using biomarkers of individual and team performance.

## Supplementary Material

## References

[1] Wood TC Raison N Haldar S . Training Tools for Nontechnical Skills for Surgeons-A Systematic Review. J Surg Educ. 2017;74:548–578. 28011262 10.1016/j.jsurg.2016.11.017

[2] Flin RH Youngson GG Yule S . Enhancing surgical performance : a primer in non-technical skills. Boca Raton, FL: Boca Raton, FL: CRC Press; 2016.

[3] Dirican AC Göktürk M . Psychophysiological measures of human cognitive states applied in human computer interaction. Procedia Comput Sci. 2011;3:1361–1367.

[4] Yu P Pan J Wang Z . Quantitative influence and performance analysis of virtual reality laparoscopic surgical training system. BMC Med Educ. 2022;22:92. 35144614 10.1186/s12909-022-03150-y PMC8832780

[5] Zhang J-Y Liu S-L Feng Q-M . Correlative Evaluation of Mental and Physical Workload of Laparoscopic Surgeons Based on Surface Electromyography and Eye-tracking Signals. Sci Rep. 2017;7:11095–11097. 28894216 10.1038/s41598-017-11584-4 PMC5594030

[6] Yule S Janda A Likosky DS . Surgical Sabermetrics: Applying Athletics Data Science to Enhance Operative Performance. Annals of Surgery Open. 2021;2:e054. 34179890 10.1097/AS9.0000000000000054 PMC8221711

[7] Dias RD Zenati MA Stevens R . Physiological synchronization and entropy as measures of team cognitive load. J Biomed Inform. 2019;96:103250–103250. 31295623 10.1016/j.jbi.2019.103250 PMC7226673

[8] Flin R O’connor P Crichton M . Safety at the sharp end: a guide to non-technical skills. CRC Press; 2017.

[9] Stanton NA Hedge A Brookhuis K . Handbook of Human Factors and Ergonomics Methods. CRC Press; 2004.

[10] Miyake S . Multivariate workload evaluation combining physiological and subjective measures. Int J Psychophysiol. 2001;40:233–238. 11228350 10.1016/s0167-8760(00)00191-4

[11] Sweller J . Cognitive load during problem solving: Effects on learning. Cogn Sci. 1988;12:257–285.

[12] Haapalainen E Kim S Forlizzi JF . Psycho-physiological measures for assessing cognitive load. Proceedings of the 12th ACM international conference on Ubiquitous computing. New York, NY: Association for Computing Machinery; 2010:301–310.

[13] Hart SG Staveland LE . Development of NASA-TLX (Task Load Index): Results of Empirical and Theoretical Research. In: Hancock PA, Meshkati N, eds. Advances in Psychology. North-Holland; 1988:139–183.

[14] Pimentel G Rodrigues S Silva PA . A wearable approach for intraoperative physiological stress monitoring of multiple cooperative surgeons. Int J Med Inform. 2019;129:60–68. 31445290 10.1016/j.ijmedinf.2019.05.028

[15] Bong CL Fraser K Oriot D . Cognitive Load and Stress in Simulation. In: Grant VJ, Cheng A, eds. Comprehensive Healthcare Simulation: Pediatrics. Cham: Springer International Publishing; 2016:3–17.

[16] Lazarus RS . Theory-based stress research. Psychol Inq. 1990;1:3–13.

[17] Schuler RS . Definition and conceptualization of stress in organizations. Organ Behav Hum Perform. 1980;25:184–215.

[18] Dias RD Ngo‐Howard MC Boskovski MT . Systematic review of measurement tools to assess surgeons’ intraoperative cognitive workload. Br J Surg. 2018;105:491–501. 29465749 10.1002/bjs.10795 PMC5878696

[19] Conway D Dick I Li Z . The Effect of Stress on Cognitive Load Measurement. Human-Computer Interaction – INTERACT 2013. Berlin Heidelberg: Springer; 2013:659–666.

[20] Anton NE Athanasiadis DI Karipidis T . Surgeon stress negatively affects their non-technical skills in the operating room. Am J Surg. 2021;222:1154–1157. 33549296 10.1016/j.amjsurg.2021.01.035

[21] Cabrera-Mino C Shinnick MA Moye S . Task-Evoked Pupillary Responses in Nursing Simulation as an Indicator of Stress and Cognitive Load. Clinical Simulation in Nursing. 2019;31:21–27.

[22] Mendes WB . Assessing autonomic nervous system activity. In: Beer J, Harmon-Jones E, eds. Methods in social neuroscience. Guilford Press; 2009:118–147.

[23] Grantcharov PD Boillat T Elkabany S . Acute mental stress and surgical performance. BJS open. 2019;3:119–125. 30734023 10.1002/bjs5.104 PMC6354185

[24] Lechappe A Chollet M Rigaud J . Assessment of Situation Awareness during Robotic Surgery using Multimodal Data. Association for Computing Machinery; 2020:412–416.

[25] Yule S Flin R Paterson-Brown S . Non-technical skills for surgeons in the operating room: a review of the literature. Surgery. 2006;139:140–149. 16455321 10.1016/j.surg.2005.06.017

[26] Cha JS Yu D . Objective Measures of Surgeon Nontechnical Skills in Surgery: A Scoping Review. Hum Factors. 2021:18720821995319–18720821995319. 10.1177/0018720821995319 33682476

[27] Tricco AC Lillie E Zarin W . PRISMA Extension for Scoping Reviews (PRISMA-ScR): Checklist and Explanation. Ann Intern Med. 2018;169:467–473. 30178033 10.7326/M18-0850

[28] Stern C Jordan Z McArthur A . Developing the Review Question and Inclusion Criteria. Am J Nurs. 2014;114:53–56. 10.1097/01.NAJ.0000445689.67800.86 24681476

[29] Howie E Wigmore SJ Dias RD . Protocol for a scoping review on ‘surgical sabermetrics:’ technology-enhanced measurement of operative non-technical skills. BMJ Open. 2023;13:e064196. 10.1136/bmjopen-2022-064196 PMC9899980 36737091

[30] Sirriyeh R Lawton R Gardner P . Reviewing studies with diverse designs: the development and evaluation of a new tool. J Eval Clin Pract. 2012;18:746–752. 21410846 10.1111/j.1365-2753.2011.01662.x

[31] Uslu S Atici Göğüs Y . Stress In the Operating Room: Emergency and Elective Surgeries. Anestezi Dergisi. 2018;26:127–131.

[32] Kennedy-Metz LR Arshanskiy M Keller S . Association between operating room noise and team cognitive workload in cardiac surgery. IEEE Conf Cogn Comput Asp Situat Manag. 2022;2022:89–93. 35984653 10.1109/cogsima54611.2022.9830675 PMC9382699

[33] Cha JS Athanasiadis DI Peng Y . Objective Nontechnical Skills Measurement Using Sensor-based Behavior Metrics in Surgical Teams. Hum Factors. 2022:187208221101292. 10.1177/00187208221101292 35610959

[34] Hall A Kawai K Graber K . Acoustic analysis of surgeons’ voices to assess change in the stress response during surgical in situ simulation. *BMJ Simulation & Technology Enhanced Learning*. 2021;7. https://access.portico.org/Portico/show?content=E-Journal%20Content&cs=ISSN_20566697_1218&auId=ark%3A%2F27927%2Fphzpr205jnv&auViewType1=PDF&auViewType2=PDF 10.1136/bmjstel-2020-000727 PMC8936944 35520977

[35] Rieger A Fenger S Neubert S . Psychophysical workload in the operating room: primary surgeon versus assistant. Surg Endosc. 2015;29:1990–1998. 25303917 10.1007/s00464-014-3899-6

[36] Presti DL Gravina R Massaroni C . A Multisensory Platform for Maximizing Collective Intelligence in the Operating Room. 2021 IEEE/ACM Conference on Connected Health: Applications. Systems and Engineering Technologies (CHASE); 2021:174–178.

[37] Dias RD Kennedy-Metz LR Yule SJ . Assessing Team Situational Awareness in the Operating Room via Computer Vision. 2022 IEEE Conference on Cognitive and Computational Aspects of Situation Management (CogSIMA). 2022:94–96. 10.1109/cogsima54611.2022.9830664 PMC9386571 35994041

[38] Wilson MR Poolton JM Malhotra N . Development and Validation of a Surgical Workload Measure: The Surgery Task Load Index (SURG-TLX). World J Surg. 2011;35:1961–1969. 21597890 10.1007/s00268-011-1141-4 PMC3152702

[39] Seelandt JC Tschan F Keller S . Assessing distractors and teamwork during surgery: developing an event-based method for direct observation. BMJ Qual Saf. 2014;23:918–929. 10.1136/bmjqs-2014-002860 25013008

[40] Sevdalis N Lyons M Healey AN . Observational teamwork assessment for surgery: construct validation with expert versus novice raters. Ann Surg. 2009;249:1047–1051. 19474694 10.1097/SLA.0b013e3181a50220

[41] Wetzel CM Black SA Hanna GB . The Effects of Stress and Coping on Surgical Performance During Simulations. Ann Surg. 2010;251:171. 20032721 10.1097/SLA.0b013e3181b3b2be

[42] Wetzel CM George A Hanna GB . Stress Management Training for Surgeons—A Randomized, Controlled, Intervention Study. Ann Surg. 2011;253:488–494. 21209585 10.1097/SLA.0b013e318209a594

[43] Phitayakorn R Minehart RD Hemingway MW . Relationship between physiologic and psychological measures of autonomic activation in operating room teams during a simulated airway emergency. Am J Surg. 2015;209:86–92. 25454964 10.1016/j.amjsurg.2014.08.036

[44] Guzmán-García C Sánchez-González P Margallo JAS . Correlating Personal Resourcefulness and Psychomotor Skills: An Analysis of Stress, Visual Attention and Technical Metrics. *Sensors* . 2022;22:837

[45] Vitous CA Dinh DQ Jafri SM . Optimizing Surgeon Well-Being: A Review and Synthesis of Best Practices. Ann Surg Open. 2021;2:e029. 36714393 10.1097/AS9.0000000000000029 PMC9872854

[46] Zenati MA Leissner KB Zorca S . First Reported Use of Team Cognitive Workload for Root Cause Analysis in Cardiac Surgery. Semin Thorac Cardiovasc Surg. 2019;31:394–396. 30578828 10.1053/j.semtcvs.2018.12.003 PMC6584063

[47] Roberts SI Cen SY Nguyen JH . The Relationship Between Technical Skills, Cognitive Workload, and Errors During Robotic Surgical Exercises. J Endourol. 2022;36:712–720. 34913734 10.1089/end.2021.0790 PMC9145254

[48] Skulmowski A Rey GD . Measuring Cognitive Load in Embodied Learning Settings. Front Psychol. 2017;8:1191. 28824473 10.3389/fpsyg.2017.01191 PMC5539229

[49] Zakeri Z Mansfield N Sunderland C . Physiological correlates of cognitive load in laparoscopic surgery. Sci Rep. 2020;10:12927. 32737352 10.1038/s41598-020-69553-3 PMC7395129

[50] Chrouser KL Xu J Hallbeck S . The influence of stress responses on surgical performance and outcomes: Literature review and the development of the surgical stress effects (SSE) framework. Am J Surg. 2018;216:573–584. 29525056 10.1016/j.amjsurg.2018.02.017

[51] Jevsevar DS Molloy IB Gitajn IL . Orthopaedic Surgeon Physiological Indicators of Strain as Measured by a Wearable Fitness Device. J Am Acad Orthop Surg. 2021;29:e1378–e1386. 33999882 10.5435/JAAOS-D-21-00078

[52] Kahol K Smith M Brandenberger J . Impact of fatigue on neurophysiologic measures of surgical residents. J Am Coll Surg. 2011;213:29–34; discussion 34-6. 21515080 10.1016/j.jamcollsurg.2011.03.028

[53] Kratzke IM Campbell A Yefimov MN . Pilot Study Using Neurofeedback as a Tool to Reduce Surgical Resident Burnout. J Am Coll Surg. 2021;232:74–80. 33022395 10.1016/j.jamcollsurg.2020.08.762

[54] Lillemoe HA Geevarghese SK . Stopping the Progression of Moral Injury: A Priority During Surgical Training. Ann Surg. 2021;274:e643–e645. 34387198 10.1097/SLA.0000000000005153

[55] Engelmann C Schneider M Kirschbaum C . Effects of intraoperative breaks on mental and somatic operator fatigue: a randomized clinical trial. Surg Endosc. 2011;25:1245–1250. 20835716 10.1007/s00464-010-1350-1

[56] Gao J Liu S Feng Q . Quantitative Evaluations of the Effects of Noise on Mental Workloads Based on Pupil Dilation during Laparoscopic Surgery. Am Surg. 2018;84:1951–1956. 30606354

[57] Yu P Pan J Wang Z . Cognitive Load/flow and Performance in Virtual Reality Simulation Training of Laparoscopic Surgery. *2021 IEEE Conference on Virtual Reality and 3D User Interfaces Abstracts and Workshops (VRW)*. 2021:466–467.

[58] Jiang X Zheng B Tien G . Pupil response to precision in surgical task execution. Stud Health Technol Inform. 2013;184:210–214. 23400158

[59] Zhang J Liu S Feng Q . Ergonomic Assessment of the Mental Workload Confronted by Surgeons during Laparoscopic Surgery. Am Surg. 2018;84:1538–1543. 30268190

[60] Stevens R Galloway T Willemsen-Dunlap A . Advancing Our Understandings of Healthcare Team Dynamics From the Simulation Room to the Operating Room: A Neurodynamic Perspective. Front Psychol. 2019;10:1660. 31456706 10.3389/fpsyg.2019.01660 PMC6699601

[61] Gunawardena N Matscheko M Anzengruber B . Assessing surgeons’ skill level in laparoscopic cholecystectomy using eye metrics. Association for Computing Machinery; 2019.

[62] Jukes AK Mascarenhas A Murphy J . Stress response and communication in surgeons undergoing training in endoscopic management of major vessel hemorrhage: a mixed methods study. Int Forum Allergy Rhinol. 2017;7:576–583. 28481016 10.1002/alr.21941

[63] Feeley AA Feeley IH McManus R . Evaluating the impact of supervision on surgical trainees stress response during simulated surgical procedures; a crossover randomized trial. *J Surg Educ* . 2022;79:1379–1386. 10.1016/j.jsurg.2022.07.006 35918278

[64] Zheng B Tien G Atkins SM . Surgeon’s vigilance in the operating room. Am J Surg. 2011;201:673–677. 21545920 10.1016/j.amjsurg.2011.01.016

[65] He W Jiang X Zheng B . Synchronization of Pupil Dilations Correlates With Team Performance in a Simulated Laparoscopic Team Coordination Task. Simul Healthc. 2021;16:e206–e213. 33534401 10.1097/SIH.0000000000000548

[66] Arora S Hull L Sevdalis N . Factors compromising safety in surgery: stressful events in the operating room. Am J Surg. 2010;199:60–65. 20103067 10.1016/j.amjsurg.2009.07.036

[67] Shafiei SB Elsayed AS Hussein AA . Evaluating the mental workload during robot-assisted surgery utilizing network flexibility of human brain. *IEEE Access* . 2020;8:204012–204019.

[68] Georgiou K Larentzakis A Papavassiliou AG . Surgeons’ and surgical trainees’ acute stress in real operations or simulation: A systematic review. Surgeon. 2017;15:355–365. 28716368 10.1016/j.surge.2017.06.003

[69] Louridas M Bonrath EM Sinclair DA . Randomized clinical trial to evaluate mental practice in enhancing advanced laparoscopic surgical performance. Br J Surg. 2015;102:37–44. 25332065 10.1002/bjs.9657

[70] Erestam S Bock D Andersson AE . The perceived benefit of intraoperative stress modifiers for surgeons: an experimental simulation study in volunteers. Patient Saf Surg. 2021;15:23–23. 34051829 10.1186/s13037-021-00294-6 PMC8164765

[71] Guru KA Esfahani ET Raza SJ . Cognitive skills assessment during robot-assisted surgery: separating the wheat from the chaff. BJU Int. 2015;115:166–174. 24467726 10.1111/bju.12657

[72] Di Stasi LL Diaz-Piedra C Rieiro H . Gaze entropy reflects surgical task load. Surg Endosc. 2016;30:5034–5043. 26983440 10.1007/s00464-016-4851-8

[73] Diaz-Piedra C Sanchez-Carrion JM Rieiro H . Gaze-based technology as a tool for surgical skills assessment and training in urology. Urology. 2017;107:26–30. 28666793 10.1016/j.urology.2017.06.030

[74] Kennedy-Metz LR Dias RD Srey R . Sensors for continuous monitoring of surgeon's cognitive workload in the cardiac operating room. Sensors. 2020;20:6616. 33227967 10.3390/s20226616 PMC7699221

[75] Dias RD Osterweil LJ Riccardi G . Development of an Interactive Dashboard to Analyze Cognitive Workload of Surgical Teams During Complex Procedural Care. IEEE International Inter-Disciplinary Conference on Cognitive Methods in Situation Awareness and Decision Support IEEE International Multi-Disciplinary Conference on Cognitive Methods in Situation Awareness and Decision Support. 2018;2018:77–82. 30547096 10.1109/COGSIMA.2018.8423995 PMC6289194

[76] Theodoraki MN Ledderose GJ Becker S . Mental distress and effort to engage an image-guided navigation system in the surgical training of endoscopic sinus surgery: a prospective, randomised clinical trial. Eur Arch Otorhinolaryngol. 2015;272:905–913. 25007736 10.1007/s00405-014-3194-0

[77] Zhang J-Y Shen Z-H Wang B-P . Influence of 3D laparoscopic surgery on surgeon’s visual pattern and mental workload. J Med Eng Technol. 2021;45:375–379. 33843431 10.1080/03091902.2021.1907466

[78] Anschuetz L Niederhauser L Wimmer W . Comparison of 3- vs 2- dimensional endoscopy using eye tracking and assessment of cognitive load among surgeons performing endoscopic ear surgery. JAMA Otolaryngol Head Neck Surg. 2019;145:838–845. 31343675 10.1001/jamaoto.2019.1765 PMC6659156

[79] Plazak J DiGiovanni DA Collins DL . Cognitive load associations when utilizing auditory display within image-guided neurosurgery. Int J Comput Assist Radiol Surg. 2019;14:1431–1438. 30997635 10.1007/s11548-019-01970-w

[80] Moore LJ Wilson MR McGrath JS . Surgeons’ display reduced mental effort and workload while performing robotically assisted surgical tasks, when compared to conventional laparoscopy. Surg Endosc. 2015;29:2553–2560. 25427414 10.1007/s00464-014-3967-y

[81] Hendy KC . Situation Awareness and Workload: Birds of a Feather? Available from: https://citeseerx.ist.psu.edu/viewdoc/summary?doi=10.1.1.492.595 Accessed August 24, 2022.

[82] Lee YH Jeon J-D Choi Y-C . Air traffic controllers’ situation awareness and workload under dynamic air traffic situations. Transp J. 2012;51:338–352.

[83] Endsley MR Garland DJ . Situation Awareness Analysis and Measurement. CRC Press; 2000.

[84] Alugubelli N Abuissa H Roka A Wearable devices for remote monitoring of heart rate and heart rate variability—what we know and what is coming. *Sensors* . 2022;22:8903. 10.3390/s22228903 PMC9695982 36433498

[85] Park B Korbach A Brünken R . Do learner characteristics moderate the seductive-details-effect? A cognitive-load-study using eye-tracking. J Educ Technol Soc. 2015;18:24–36.

[86] Tricco AC Lillie E Zarin W . A scoping review on the conduct and reporting of scoping reviews. BMC Med Res Methodol. 2016;16:15. 26857112 10.1186/s12874-016-0116-4 PMC4746911

[87] Boucsein W . Electrodermal Activity. Springer Science & Business Media; 2012.

[88] van Houwelingen BCG Rutkowski A-F Ganni S . Effects of surgical flow disruptions on surgeons’ resources: a pilot study. Surg Endosc. 2020;34:4525–4535. 31720810 10.1007/s00464-019-07239-2

[89] Modi HN Singh H Fiorentino F . Association of residents' neural signatures with stress resilience during surgery. JAMA Surg. 2019;154:e192552. 31389994 10.1001/jamasurg.2019.2552 PMC6686757

[90] Gao J Liu S Feng Q . Subjective and objective quantification of the effect of distraction on physician's workload and performance during simulated laparoscopic surgery. Med Sci Monit. 2019;25:3127–3132. 31030208 10.12659/MSM.914635 PMC6503751

[91] Shafiei SB Iqbal U Hussein AA . Utilizing deep neural networks and electroencephalogram for objective evaluation of surgeon’s distraction during robot-assisted surgery. Brain Res. 2021;1769:147607. 34352240 10.1016/j.brainres.2021.147607

[92] Habibi E Taheri MR Hasanzadeh A . Relationship between mental workload and musculoskeletal disorders among Alzahra Hospital nurses. Iran J Nurs Midwifery Res. 2015;20:1–6. 25709683 PMC4325400

[93] Bolghanabadi S Bolghanabadi N Mosavianasl Z . Relationship between mental workload‌ ‌and musculoskeletal disorders in nurses working at day and night shifts in the state hospitals. Int J Musculoskelet Pain Prev. 2018;3:7–11.

[94] Mahmoudifar Y Seyedamini B . Investigation on the relationship between mental workload and musculoskeletal disorders among nursing staff. International Archives of Health Sciences. 2018;5:16.

[95] Heidarimoghadam R Saidnia H Joudaki J . Does mental workload can lead to musculoskeletal disorders in healthcare office workers? Suggest and investigate a path. Cogent Psychology. 2019;6:1664205.

[96] Deeney C O’Sullivan LW . Effects of cognitive loading and force on upper trapezius fatigue. Occup Med. 2017;67:678–683. 10.1093/occmed/kqx157 29165609

[97] Biondi FN Cacanindin A Douglas C . Overloaded and at work: investigating the effect of cognitive workload on assembly task performance. Hum Factors. 2021;63:813–820. 32530759 10.1177/0018720820929928 PMC8273843

[98] Grant KMK Vo T Tiong LU . The painful truth: work-related musculoskeletal disorders in Australian surgeons. Occup Med. 2020;70:60–63. 10.1093/occmed/kqz155 31829426

[99] Chambers A Gill N . Work related musculoskeletal pain in general surgical trainees: extent of the problem and strategies for injury prevention. Bull R Coll Surg Engl. 2020;102:e9–e14.

[100] Poolton JM Wilson MR Malhotra N . A comparison of evaluation, time pressure, and multitasking as stressors of psychomotor operative performance. Surgery. 2011;149:776–782. 21310451 10.1016/j.surg.2010.12.005

[101] Erridge S Ashraf H Purkayastha S . Comparison of gaze behaviour of trainee and experienced surgeons during laparoscopic gastric bypass. Br J Surg. 2018;105:287–294. 29193008 10.1002/bjs.10672

[102] Wright MC Taekman JM Endsley MR . Objective measures of situation awareness in a simulated medical environment. Qual Saf Health Care. 2004;13(Suppl 1):i65–71. 15465958 10.1136/qshc.2004.009951 PMC1765787

[103] Pluyter JR Rutkowski A Jakimowicz J . Immersive training: breaking the bubble and measuring the heat. Surg Endosc. 2013;28:1545–1554. 10.1007/s00464-013-3350-4 24399519

[104] Manzey D Luz M Mueller S . Automation in surgery: the impact of navigated-control assistance on performance, workload, situation awareness, and acquisition of surgical skills. Hum Factors. 2011;53:584–599. 22235522 10.1177/0018720811426141

[105] Kennedy-Metz L Dias R Zenati M . The Cognitive Relevance of a Formal Pre-incision Time-out in Surgery. Proceedings of the 32nd European Conference on Cognitive Ergonomics (ECCE ’21). New York, NY: Association for Computing Machinery; 2021:1–5. 10.1145/3452853.3452867 PMC8528342 34676380

[106] Kennedy-Metz LR Dias RD Stevens RH . Analysis of mirrored psychophysiological change of cardiac surgery team members during open surgery. J Surg Educ. 2021;78:622–629. 32863172 10.1016/j.jsurg.2020.08.012 PMC7904574

[107] Pugh CM Santacaterina S DaRosa DA . Intra-operative decision making: more than meets the eye. J Biomed Inform. 2011;44:486–496. 20096376 10.1016/j.jbi.2010.01.001

[108] Pignatiello GA Martin RJ Hickman RL Jr . Decision fatigue: A conceptual analysis. J Health Psychol. 2020;25:123–135. 29569950 10.1177/1359105318763510 PMC6119549

[109] Price T Tenan M Head J, Acute Stress Causes Over Confidence in Situation Awareness. *2016 IEEE International Multi-Disciplinary Conference on Cognitive Methods in Situation Awareness and Decision Support (CogSIMA).* ieeexplore.ieee.org; 2016:1–6.

[110] Wu C Cha J Sulek J . Sensor-based indicators of performance changes between sessions during robotic surgery training. Appl Ergon. 2021;90:103251. 32961465 10.1016/j.apergo.2020.103251 PMC7606790

[111] Shafiei SB Jing Z Attwood K . Association between functional brain network metrics and surgeon performance and distraction in the operating room. Brain Sci.. 2021;11:468. 33917719 10.3390/brainsci11040468 PMC8068138

[112] Suarez-Revelo JX Ochoa-Gomez JF Hernandez-Valdivieso AM . Neurophysiological changes associated with training in laparoscopic surgery using EEG: a pilot study. Conf Proc IEEE Eng Med Biol Soc. 2019;2019:4572–4575. 10.1109/EMBC.2019.8856980 31946882

[113] James OP Robinson DBT Hopkins L . Biosensors, biomarkers and biometrics: A bootcamp perspective. BMJ Simul Technol Enhanc Learn. 2020: bmjstel-2020-000631. 10.1136/bmjstel-2020-000631 PMC8936825 35516828

[114] Sweller J . Cognitive load theory and educational technology. Educ Technol Res Dev. 2020;68:1–16.

[115] Shaker D . Cognitivism and psychomotor skills in surgical training: from theory to practice. J Int Assoc Med Sci Educ. 2018;9:253–254. 10.5116/ijme.5b9a.129b PMC6387771 30269109

[116] Howie EE Dharanikota H Gunn E . Cognitive load management: an invaluable tool for safe and effective surgical training. J Surg Educ. 2023;80:311–322. 36669990 10.1016/j.jsurg.2022.12.010

[117] Maimon NB Bez M Drobot D . Continuous monitoring of mental load during virtual simulator training for laparoscopic surgery reflects laparoscopic dexterity. A comparative study using a novel wireless device. Front Neurosci. 2022;15:1716. 10.3389/fnins.2021.694010 PMC8811150 35126032

[118] Kneebone RL Scott W Darzi A . Simulation and clinical practice: strengthening the relationship. Med Educ. 2004;38:1095–1102. 15461655 10.1111/j.1365-2929.2004.01959.x

[119] Shafiei SB Hussein AA Muldoon SF . Functional brain states measure mentor-trainee trust during robot-assisted surgery. Sci Rep. 2018;8:3667–3612. 29483564 10.1038/s41598-018-22025-1 PMC5827753

[120] Ji Y Kong Z Deng Y . The role of eye tracker in teaching video-assisted thoracoscopic surgery: the differences in visual strategies between novice and expert surgeons in thoracoscopic surgery. Ann Transl Med. 2022;10:592. 35722359 10.21037/atm-22-2145 PMC9201158

[121] Wilson C Chahine S Cristancho S . Unusual suspects: real-time physiological evaluation of stressors during laparoscopic donor nephrectomy. Can Urol Assoc J. 2021;15:E205–E209. 33007178 10.5489/cuaj.6647 PMC8021419

[122] Modi HN Singh H Orihuela-Espina F . Temporal stress in the operating room: brain engagement promotes “coping” and disengagement prompts “choking”. Ann Surg. 2018;267:683–691. 28489681 10.1097/SLA.0000000000002289

[123] Modi HN Singh H Darzi A . Multitasking and time pressure in the operating room: impact on surgeons’ brain function. Ann Surg. 2020;272:648. 32657937 10.1097/SLA.0000000000004208

[124] Wetzel CM Kneebone RL Woloshynowych M . The effects of stress on surgical performance. Am J Surg. 2006;191:5–10. 16399098 10.1016/j.amjsurg.2005.08.034

[125] Kahneman D . Thinking, fast and slow. United States: Farrar, Straus and Giroux; 2012.

[126] Menekse Dalveren GG Cagiltay NE . Distinguishing intermediate and novice surgeons by eye movements. Front Psychol. 2020;11:542752. 33013592 10.3389/fpsyg.2020.542752 PMC7511664

[127] Charles RL Nixon J . Measuring mental workload using physiological measures: a systematic review. Appl Ergon. 2019;74:221–232. 30487103 10.1016/j.apergo.2018.08.028

[128] Zheng B Jiang X Tien G . Workload assessment of surgeons: correlation between NASA TLX and blinks. Surg Endosc. 2012;26:2746–2750. 22527300 10.1007/s00464-012-2268-6

[129] Morales JM Ruiz-Rabelo JF Diaz-Piedra C . Detecting mental workload in surgical teams using a wearable single-channel electroencephalographic device. J Surg Educ. 2019;76:1107–1115. 30691989 10.1016/j.jsurg.2019.01.005

[130] Duru DG Deniz Duru A Barkana DE . Assessment of surgeon’s stress level and alertness using EEG during laparoscopic simple nephrectomy. *2013 6th International IEEE/EMBS Conference on Neural Engineering (NER)*. 2013.

[131] Guru KA Shafiei SB Khan A . Understanding Cognitive Performance During Robot-Assisted Surgery. Urology. 2015;86:751–757. 26255037 10.1016/j.urology.2015.07.028

[132] Tien T Pucher PH Sodergren MH . Differences in gaze behaviour of expert and junior surgeons performing open inguinal hernia repair. Surg Endosc. 2015;29:405–413. 25125094 10.1007/s00464-014-3683-7

[133] Kramer AF . Physiological metrics of mental workload: A review of recent progress. *Multiple-task Performance*. 2020. DOI: 10.1201/9781003069447-14/physiological-metrics-mental-workload-review-recent-progress-arthur-kramer

[134] Grassmann M Vlemincx E von Leupoldt A . Respiratory changes in response to cognitive load: a systematic review. Neural Plast. 2016;2016:8146809–8146816. 27403347 10.1155/2016/8146809 PMC4923594

[135] Adams CE Leverland MB . Environmental and behavioral factors that can affect blood pressure. Nurse Pract. 1985;10:39–40. 10.1097/00006205-198511000-00005 4069459

[136] Hurley AM Kennedy PJ O’Connor L . SOS save our surgeons: Stress levels reduced by robotic surgery. Gynecol Surg. 2015;12:197–206.

[137] Tao D Tan H Wang H . A systematic review of physiological measures of mental workload. Int J Environ Res Public Health. 2019;16:2716. 31366058 10.3390/ijerph16152716 PMC6696017

[138] Levin M McKechnie T Kruse CC . Surgical data recording in the operating room: a systematic review of modalities and metrics. Br J Surg. 2021;108:613–621. 34157080 10.1093/bjs/znab016

[139] Agha RA Fowler AJ Sevdalis N . The role of non-technical skills in surgery. Ann West Med Surg. 2015;4:422–427. 10.1016/j.amsu.2015.10.006 PMC4720712 26904193

[140] Howie E Wigmore S Skipworth R . Development of a surgical sabermetrics model. Health Research Authority. 2022. Accessed January 19, 2023. https://www.hra.nhs.uk/planning-and-improving-research/application-summaries/research-summaries/development-of-a-surgical-sabermetrics-model/

