# Supplementary material for: Surgical Sabermetrics: A Scoping Review of Technology-enhanced Assessment of Nontechnical Skills in the Operating Room
Source: Ann Surg. 2024 Jan 23;279(6):973–84. doi: 10.1097/SLA.0000000000006211 (PMC11086675; doi:10.1097/SLA.0000000000006211)
Supplement: Supplementary file 1 [file sla-279-0973-s001.docx]

# Supplemental Data

This content contains the following additional data for this scoping review:

- Supplemental Table 1: Data extraction from all studies included.
- Supplemental Table 2. Mapping Sabermetric concepts and biomarkers against NOTSS categories.
- Appendixes A-C

Supplemental Table 1: Data extraction from all studies included, organised by operative (1a) and simulation (1b) studies.

| **1a. Operative studies** | | | | | | | | | | | |
| --- | --- | --- | --- | --- | --- | --- | --- | --- | --- | --- | --- |
| **Paper** | **Location** | **Speciality** | **Purpose** | **Concepts/Aim of Study** | **Participants** | **Surgery Modality** | **Non-Technical skill** | **Objective Metric** | **Subjective Metric** | **QATSDD**  **%**  **(1-100%)** | **Results/Conclusions** |
| Wilson et al. 2021. | Canada | Urology | 1. Measure task demands 2. Quantify expertise | To investigate EDA during surgery to determine the effect of case difficulty, distractions, and learner expertise | n = 1  Fully Qualified Surgeon | Laparoscopic | Stress  Situation Awareness | EDA |  | 57% | EDA changes suggested the significant impact of learner autonomy and case difficulty on trainers’ stress response and Situation Awareness |
| Gunawardena et al. 2019. | Austria | General Surgery | 1. Quantify expertise | To investigate how eye metrics can reflect the expertise levels of surgeons | n = 4  Surgeons (Mixed ability - unspecified) | Laparoscopic | Cognitive Load | Eye-metrics: Pupillometry Eye-tracking |  | 67% | Pupillary metrics can identify changes to Cognitive Load. Eye-metrics can differentiate skill levels. |
| Grantcharov et al. 2019. | USA | Bariatrics | Examine performance modulation | To investigate the association between acute intraoperative mental stress and technical surgical performance | n = 1  Fully Qualified Surgeons | Laparoscopic | Stress | HRV |  | 65% | There is an association between measures of acute mental stress and worse technical surgical performance |
| Erridge et al. 2018. | UK | Bariatrics | 1. Quantify expertise | To identify differences in gaze behaviour, cognitive attention, and workload between expert and junior surgeons | n = 4  Surgeons (Mixed ability - unspecified) | Laparoscopic | Cognitive Load  Situation Awareness | Eye-metrics: Eye-tracking, Blink rate |  | 64% | Expert surgeons exert attentional focus on significant stimuli with reduced Cognitive Load and increased concentration compared to novice surgeons |
| Ji et al. 2022 | China | Cardiothoracic | Evaluate training efficacy | To investigate if an expert surgeon’s eye movement tracking can improve learning by novice surgeons | n = 16  Trainee Surgeons (8), Fully Qualified Surgeons (8) | Laparoscopic | Cognitive Load | Eye-Metrics: Eye-Tracking |  | 64% | Novice and expert surgeons have different eye fixation patterns, search strategies and Cognitive Load patterns |
| Engelmann et al. 2011. | Germany | General Surgery, Urology | Evaluate a non-training intervention | To evaluate the effects of intraoperative breaks on the surgeon | n = 7  Fully Qualified surgeons | Laparoscopic | Cognitive Load | HRV | NASA-TLX derivatives | 58% | Breaks during complex surgery can reduce psychological stress and Cognitive Load while preserving performance without prolongation of the operation time |
| Ducarme et al. 2015. | France | Obstetrics and Gynaecology | Stress quantification | To describe heart rate variations in surgical residents to assess their intraoperative stress | n = 75  Trainee Surgeons | Laparoscopic | Stress | HR |  | 52% | HR fluctuations reflected potential stressors during surgery |
| Duru et al. 2013. | Turkey | Urology | Measure task demands | To monitor and utilize objective information on the mental effort and stress demanded in surgeons | n = 1  Fully Qualified Surgeon | Laparoscopic | Stress  Situation Awareness | EEG |  | 48% | The most stressful surgical stage can be identified using EEG, allowing surgeons to seek help to decrease their level of stress in this phase |
| Kennedy-Metz et al. 2021. | USA | Cardiothoracic | Team performance assessment | To investigate if individual cognitive load reveals team workload states during pre-incision time-out | n = 2  Fully Qualified Surgeons (as part of mixed profession team) | Open | Cognitive Load  Teamwork | HRV |  | 52% | Lack of HRV and Cognitive Load changes amongst team members provide objective evidence of shared mental model and effective teamwork via psychophysiological mirroring |
| Tien et al. 2015. | USA | General Surgery | 1. Quantify expertise | To assess differences in gaze behaviour between experts and juniors | n = 9  Surgeons (Mixed ability - unspecified) | Open | Cognitive Load | Eye-metrics: Pupillometry Eye-tracking | NASA-TLX | 42% | Eye-tracking technology is feasible for skill assessment and can differentiate between expertise levels. Eye-metrics can identify Cognitive Load fluctuations. |
| Kennedy-Metz et al. 2020. | USA | Cardiothoracic | User testing in technological innovation | To investigate the feasibility of collecting multi-modal psychophysiological inputs in the real-world setting of the OR | n = 1  Fully Qualified Surgeon (as part of mixed profession team) | Open | Cognitive Load | fNIRS  HRV |  | 60% | Changes in Cognitive Load metrics correlated with intra-operative events across surgical phases |
| Zenati et al. 2019. | USA | Cardiothoracic | Analyze patient safety | To presents a case of a near-miss (medication error) that underwent root cause analysis using workload data | n = 1  Fully Qualified Surgeon (as part of mixed profession team) | Open | Cognitive Load | HRV |  | 43% | An episode of cognitive overload was associated with a preventable error demonstrating the utility of Cognitive Load measurements in reflection and potential error prevention |
| Pimentel et al. 2019. | Portugal | Neurosurgery | Stress quantification | To evaluate surgeons' stress levels during intracranial aneurysm procedures | n = 2  Fully Qualified Surgeons | Open  (Neurosurgery) | Cognitive Load  Stress | HRV | SURG-TLX | 67% | Cognitive Load was positively related with stress, suggesting that more cognitively demanding procedures are also more stressful |
| Kennedy-Metz et al. 2021. | USA | Cardiothoracic | Team performance assessment | To investigate the frequency of mirrored changes, defined as concurrent peaks in HRV across team members | n = 3  Fully Qualified Surgeon (as part of mixed profession team) | Open | Cognitive Load  Teamwork | HRV |  | 69% | Mirrored physiological responses representing peaks in Cognitive Load were observed during technically challenging surgical phases suggestive of teamwork quality |
| Dias et al. 2018. | USA | Cardiothoracic | Team performance assessment  User testing in technological innovation | To integrate objective measures of team member cognitive load with procedural, behavioural, and contextual data | n = 1  Fully Qualified Surgeon (as part of mixed profession team) | Open | Cognitive Load  Teamwork | HRV |  | 81% | Integration of multiple data sources is feasible and enabled detection of Cognitive Load fluctuations detection in response to tasks |
| Dias et al. 2022 | USA | Cardiothoracic | User testing in technological innovation | To investigate the feasibility of using computer vision for motion detection to identify team situation awareness | n= 14  Fully Qualified Surgeons (as part of mixed profession team) | Open | Situation Awareness  Teamwork | Movement (Computer Vision) | NOTSS | 64% | It is feasible to use surgical team motion metrics to distinguish surgical teams with good and poor Situation Awareness |
| Kennedy-Metz et al. 2022 | USA | Cardiothoracic | Team performance assessment  Examine performance modulation | To investigate team cognitive workload levels and communication in relation to peaks in ambient noise | n = 3  Fully Qualified Surgeons (as part of mixed profession team) | Open | Cognitive Load  Situation Awareness Teamwork  Communication | Acoustic Analysis  HR | Behavioural rating scale^114^ | 64% | Case-irrelevant conversations associated with a greater degree of excessive peaks in noise are associated with team Cognitive Load levels, suggestive of the need to standardize communication during critical surgical phases |
| Dias et al. 2019. | USA | Cardiothoracic | User testing in technological innovation | To establish the feasibility of using HRV for physiological synchronization analysis and objective measurement of team cognition | n = 1  Fully Qualified Surgeon (as part of mixed profession team) | Open | Cognitive Load  Teamwork | HRV |  | 64% | Physiological synchronization via HRV can provide evidence of teamwork and cognition |
| Kelkar et al. 2021 | India | Ophthalmology | Measure task demands | To investigate Cognitive Load, complication rates and visual outcomes in different methods of cataract surgery | n = 3  Fully Qualified Surgeons | Open (microscopic) | Cognitive Load | HR  SpO2 | SURG-TLX | 67% | Newer surgical systems are non-inferior regarding Cognitive Load, duration of surgery, complication rates, and patient outcomes |
| Theodoraki et al. 2015. | Germany | ENT | Measure task demands  Compare different surgical modalities | To investigate the mental and physical demands during trans nasal surgery with and without the aid of a navigation system | n= 10  Trainee Surgeon (8), Fully Qualified Surgeon (2) | Endoscopic | Cognitive Load | HR  HRV  Masticator tone (EMG)  RR |  | 60% | Cognitive Load is raised during trans nasal surgery compared to the baseline but when using navigation, it did not significantly differ |
| Shafiei et al. 2018. | USA | Urology | Evaluate training efficacy | To develop a novel method of objectively assessing mentor-trainee trust based on patterns of brain activity | n = 4  Trainee Surgeon (3), Fully Qualified Surgeon (1) | Robotic | Cognitive Load  Teamwork Leadership Situation Awareness | EEG | NASA TLX | 55% | Patterns of brain activity via EEG can objectively measure skills relating to trainee mentorship |
| Guru et al. 2015. | USA | Urology | Measure task demands | To understand cognitive function in various surgical scenarios while performing robot-assisted surgery | n = 10  Surgeons (Mixed ability - unspecified) | Robotic | Cognitive Load  Situation Awareness | EEG | NASA TLX | 33% | Cognitive analysis via EEG measurements illustrates the utilisation of cognitive resources in response to demand |
| Lechappe et al. 2020. | Netherlands | Urology | User testing in technological innovation | To propose a multimodal method for the assessment of SA | Undeclared  Fully Qualified Surgeons (as part of mixed profession team) | Robotic | Cognitive Load  Stress  Situation Awareness | EDA  HR  HRV | RTLX | 35% | Physiological measurements can be used for behavioural assessment including of Situation Awareness, Cognitive Load and stress |
| Shafiei, et al. 2021. | USA | Urology | Evaluate training efficacy  Individual performance assessment | To examine EEG features to evaluate trainee performance and distraction | n= 3  Trainee Surgeons | Robotic | Situation Awareness  Cognitive Load | EEG | NASA-TLX and SURG-TLX | 55% | Changes to EEG features during surgery related to performance and distraction |
| Hussein et al. 2016. | USA | Urology | Evaluate training efficacy | To investigate cognitive workload assessments and mentorship | n = 1  Fully Qualified Surgeon | Robotic | Cognitive Load  Situation Awareness  Leadership | EEG | NASA-TLX | 57% | Mentors Cognitive Load and Situation Awareness increased when they subjectively felt the trainee’s cognitive demand and effort were low |
| Cha et al. 2022 | USA | General Surgery, Urology | Team Performance assessment | To identify objective measures that predict surgeon nontechnical skills during surgery | n = 16  Trainee Surgeons (12), Fully Qualified Surgeons (4) (as part of mixed profession team) | Robotic | Teamwork Communication | Acoustic Analysis  Movement | NOTSS | 50% | Sensor-based measures can potentially predict NOTSS scores which can be utilized for reducing resources for performance assessment |
| Jevsevar et al. 2021. | USA | Orthopaedics | Measure task demands  Support provider wellbeing | To investigate real-time measurements of physiological strain and identify potential daily stressors | n = 21  Trainee Surgeon (12), Fully Qualified Surgeon (9) | Mixture (not specified) | Cognitive Load | HRV |  | 73% | Real-time physiological measurements can identify stressors affecting Cognitive Load, and those at risk of burnout to target intervention |
| Engelmann et al. 2014 | Germany; Switzerland | Paediatric Surgery | Evaluate a non-training intervention | To assess the impact of a noise-reduction program in a pediatric operating theatre | n = 16  Fully Qualified Surgeon | Mixture (not specified) | Cognitive Load  Situation Awareness | EDA |  | 69% | Noise-reduction was associated with a significantly lower number of complications and marked improvements in Cognitive Load |
| Uslu et al. 2018. | Turkey | Undeclared | Team performance assessment | To evaluate the level and effect of stress on teamwork in operating rooms, by utilizing quantitative and qualitative assessment | Undeclared  Surgeon (Unknown ability, as part of mixed profession team) | Mixture (not specified) | Stress  Teamwork | HR | OTAS | 33% | Team members exhibit different stress levels at different times. Stress is highest in emergencies. Variable and elevated stress levels negatively affect teamwork |
| Arora et al. 2010. | UK; Australia | General Surgery, Cardiothoracic,Orthopedics | User testing in technological innovation | To investigate the feasibility, reliability, and validity of the Imperial Stress Assessment Tool to measure stress during surgery | n = 11  Surgeons (Mixed ability - unspecified) | Mixture (open and laparoscopic) | Stress | HRV |  | 69% | Combining subjective and objective methods for measuring stress in the operating room is feasible and nonintrusive |
| van Houwelingen et al. 2020. | Netherlands | General Surgery | Measure task demands  Examine performance modulation | To evaluate the impact of disruptions and interruptions on stress level, physiological resources, and performance | n = 8  Surgeons (Mixed ability - unspecified) | Not specified | Cognitive Load  Situation Awareness | EDA  METs  Temperature |  | 43% | Variations in Cognitive Load due to factors affecting Situation Awareness are more pronounced in novice surgeons compared to expert. Disruptions affect mental, emotional and physiological resources |
| Rieger et al. 2015. | Germany | Not declared ("Surgical disciplines") | Cognitive Load quantification | To assess individual workload by combining objective and subjective measures | n = 25  Surgeons (Mixed ability - unspecified) | Not specified | Cognitive Load | HR  Movement  RR Temperature | NASA-TLX | 46% | Changes to Cognitive Load relate to surgery duration not operative role |
| Stevens et al. 2019. | USA | Neurosurgery | Team performance assessment  Compare different surgical modalities | To determine if teams show similar EEG changes performing a surgery compared to simulation | n = 3  (Unknown ability, as part of mixed profession team) | Not specified | Teamwork | EEG |  | 31% | Team members EEG changes were similar in both the simulation and live patient environments |
| **1b. Simulation studies** | | | | | | | | | | | |
| **Paper** | **Location** | **Speciality** |  | **Concepts/Aim of Study** | **Participants** | **Surgery Modality** | **Non-Technical skill** | **Objective Metric** | **Subjective Metric** | **QATSDD**  **%**  **(1-100%)** | **Results/Conclusions** |
| Suarez-Revelo et al. 2019. | Columbia | Not declared (Laparoscopic Tasks) | Evaluate training efficacy | To evaluate the effect of a training session on task performance and mental workload | n = 8 Bio-  engineering Students | Laparoscopic | Cognitive Load | EEG | NASA-TLX | 52% | Evidence of changes in Cognitive Load and improvements in task performance were associated with the training session |
| Zhang et al. 2017. | China | General Surgery | Cognitive Load quantification  Measure task demands | To measure the differences of cognitive load across phases of surgery | n = 14  Fully Qualified Surgeons (4),  Medical Students (10) | Laparoscopic | Cognitive Load | Eye-metrics: Eye-tracking  EMG |  | 38% | Evidence of varying Cognitive Load across different surgical phases. Cognitive Load and physical load appear non-synchronous |
| Zhang et al. 2021. | China | Not declared (Laparoscopic Tasks) | Compare different surgical modalities | To investigate cognitive load and visual search efficiency in 2D v 3D surgery | n = 10  Surgeons (Mixed ability - unspecified) | Laparoscopic | Cognitive Load | Eye-metrics: Eye-tracking |  | 36% | Surgeons Cognitive Load in 3D laparoscopic surgery is low compared to traditional methods |
| Kahol et al. 2011. | USA | Not declared (Laparoscopic Tasks) | Support provider wellbeing  Examine performance modulation | To gain insight into the impact of fatigue on proficiency, behavioural and neurophysiologic measures | n = 7  Trainee Surgeons | Laparoscopic | Cognitive skills  Cognitive Load  Situation Awareness | EEG |  | 60% | Reduced proficiency and attention plus increased distraction, cognitive errors and Cognitive Load are evident on EEG in fatigued residents |
| Modi et al. 2019. | UK | General Surgery | Examine performance modulation | To compare neurological measurements between surgeons demonstrating stable and stress-related performance decline | n = 33  Trainee Surgeons | Laparoscopic | Cognitive Load  Stress  Situation Awareness | fNIRS  HRV | SURG-TLX | 57% | fNIRS can demonstrate Cognitive Load changes and executive brain function including attention and concentration |
| Poolton et al. 2011. | Hong Kong; UK | Not declared (Laparoscopic Tasks) | Examine performance modulation | To examine the influence of different sources of stress on trained laparoscopic performance | n = 30  Medical Students | Laparoscopic | Stress | HR |  | 52% | Stress metrics do not reflect the demands of an operative task, highlighting the need to better understand mechanisms that influence performance |
| Modi et al. 2018. | USA | General Surgery | Examine performance modulation Measure task demands | To investigate the impact of time pressure on fNIRS measurements and performance | n = 33  Trainee Surgeons | Laparoscopic | Cognitive Load | fNIRS  HRV | SURG-TLX | 71% | Performing tasks under demand leads to prefrontal attenuation and “deactivation” on fNIRS which is associated with increased Cognitive Load |
| Pluyter et al. 2013. | Netherlands | General Surgery | Measure task demands | To investigate mental strain and cognitive absorption during a virtual reality laparoscopic simulator | n = 21  Trainee Surgeons | Laparoscopic | Cognitive Load  Situation Awareness | EDA  Temperature |  | 38% | Physiological measures of Cognitive Load, concentration and attentional focus during surgical simulation are feasible |
| Louridas et al. 2015. | Canada | Bariatrics | Evaluate a non-training intervention | To develop mental practice scripts and to assess their effect on skills and stress levels in a crisis scenario | n = 20  Trainee Surgeons | Laparoscopic | Stress | BP  HR | NOTSS | 60% | There were no differences in objective or subjective stress levels as measured by HR and BP. An improvement in technical skill was seen |
| Jiang et al. 2013. | Canada | Not declared (Laparoscopic Tasks) | Measure task demands | To explored changes in mental workload over different surgical tasks requiring increasing precision | n = 12  Surgeons (Mixed ability - unspecified), Office staff | Laparoscopic | Cognitive Load | Eye-metrics: Pupillometry |  | 48% | The rate of change of pupil diameter matches the demand of a surgical task and acts as a key behavioural indicator for assessing Cognitive Load |
| Zhang et al. 2018. | China | Not declared (Laparoscopic Tasks) | Measure task demands | To assess if cognitive load change due to different tasks is reflected in physiology | n = 16  Surgeons (Mixed ability - unspecified), Medical students | Laparoscopic | Cognitive Load | Eye-metrics: Eye-tracking | NASA-TLX | 83% | Cognitive Load variation is dependent on varying task demands |
| Gao et al. 2018. | China | General Surgery | Measure task demands | To investigate the effect of noise and therefore distraction on cognitive load during surgery | n = 24  Trainee Surgeons | Laparoscopic | Cognitive Load  Situation Awareness | Eye-metrics: Pupillometry Eye-tracking | NASA-TLX | 57% | Noise distractions are associated with significant increases in pupil sizes and Cognitive Load which may affect surgical performance |
| Modi et al. 2020. | UK | Not declared (Laparoscopic Tasks) | Measure task demands | To assess the impact of multitasking and time pressure on surgeons’ brain function during laparoscopic suturing | n = 29  Trainee Surgeons | Laparoscopic | Cognitive Load  Cognitive skills | fNIRS  HR | SURG-TLX | 71% | Performance degradation during high Cognitive Load conditions is associated with deactivation of prefrontal regions important for attentional control, working memory, and cognitive flexibility |
| Gao et al. 2019. | China | General Surgery | Measure task demands | To investigate the effect of additional cognitive tasks on cognitive load by measuring eye-metrics and subjective assessments | n = 24  Trainee Surgeons | Laparoscopic | Cognitive Load  Situation Awareness | Eye-metrics: Pupillometry Eye-tracking (Gaze) | NASA-TLX | 60% | Distractions increase the psychological burden and Cognitive Load while affecting their operational skills, threatening patient safety |
| Di Stasi et al. 2016. | Spain; USA | Not declared (Laparoscopic Tasks) | Measure task demands  Technical development | To measure task overload imposition and to investigate the use of gaze metrics to measure task load variations | n = 18  Trainee Surgeons | Laparoscopic | Cognitive Load | Eye-metrics: Eye-tracking | NASA-TLX | 76% | Eye-metrics changed in response to increased task complexity and provided an accurate marker of increased Cognitive Load |
| He et al. 2021. | Canada | Not declared (Laparoscopic Tasks) | Team performance assessment | To investigate team members' shared pupil dilations as a surrogate for team performance | n = 14  Trainee Surgeon (2), Fully Qualified Surgeon (12) | Laparoscopic | Cognitive Load  Situation Awareness  Teamwork | Eye-metrics: Pupillometry Eye-tracking |  | 74% | Pupil dilations of higher performance teams were more synchronized than those of lower performance teams suggesting shared mental model in the higher performing teams |
| Erestam et al. 2021. | Sweden | General Surgery | Measure task demands  Evaluate a non-training intervention | To evaluate simulated intraoperative stressors on surgeons’ stress levels and the effect of an intervention | n = 17  Surgeons (Mixed ability - unspecified) | Laparoscopic | Stress | HRV |  | 55% | The surgeons’ experience of a break was positive but there were no differences in physiological outcomes of the intervention |
| Zheng et al. 2012. | Canada | General Surgery | User testing in technological innovation | To investigate the correlation between subjective objective cognitive load measurements | n = 23  Surgeons (Mixed ability - unspecified) | Laparoscopic | Cognitive Load | Eye-metrics: Blink rate | NASA-TLX | 64% | Changes to eye-metrics correlated with increasing Cognitive Load |
| Zakeri et al. 2020. | UK | Not declared (Laparoscopic Tasks) | User testing in technological innovation | To assess cognitive load via physiological measurements during surgery | n = 31  Medical Students | Laparoscopic | Cognitive Load | HRV  Eye-metrics: Blink rate  fNIRS | NASA-TLX | 71% | Multi-modal physiological measurements are better predictors than any individual metric of Cognitive Load |
| Yu P et al. 2021. | China | Not declared (Virtual Reality Laparoscopic Tasks) | Measure task demands  Evaluate training efficacy  Individual performance assessment | To investigate the cognitive load, flow, and performance in immersive virtual reality simulation training of laparoscopic surgery | n = 41  Medical Students | Laparoscopic (Virtual Reality) | Cognitive Load | EEG  HR | NASA-TLX | 25% | Immersive Virtual Reality simulation training increased Cognitive Load and decreased performance compared to conventional Virtual Reality |
| Yu et al. 2022 | China | Not declared (Laparoscopic Tasks) | Measure task demands  Examine performance modulation | To investigate virtual reality simulation and its effect on performance, flow and Cognitive Load | n = 51  Medical Students | Laparoscopic (Virtual Reality) | Cognitive Load | EEG  HR | NASA-TLX | 69% | Participants’ performance is negatively correlated with Cognitive Load through quantitative, objective physiological analysis |
| Zheng et al. 2011 | Canada | General Surgery | Individual performance assessment | To assess surgeon's vigilance of the patient's condition | n = 23  Trainee Surgeons (10), Fully Qualified Surgeons (13) | Laparoscopic | Situation Awareness | Eye-metrics: Eye-tracking | NASA-TLX | 55% | Eye-tracking observations can lead to inferences regarding a surgeon's behavior for patient safety |
| Malmon et al. 2021 | Israel | Not declared (Laparoscopic Tasks) | Evaluate training efficacy | To measure the Cognitive Load of trainee surgeons to determine whether they are ready to take part in a real surgery | n = 48  (Unspecified. Unexperienced Trainee Surgeons, Medical Students) | Laparoscopic | Cognitive Load | EEG |  | 79% | EEG can demonstrate lower Cognitive Load and higher performance and be used to assess Cognitive Load and the efficacy of simulations |
| Guzmán-García et al. 2022 | Spain | Not declared (Laparoscopic Tasks) | Evaluate training efficacy | To assess personal resourcefulness by monitoring parameters relating to stress and visual attention and whether there is a relation with psychomotor skills in surgical education | n = 8  Trainee Surgeons (6), Fully Qualified Surgeons (2) | Laparoscopic | Stress  Situation Awareness | EDA  Eye-metrics: Eye-tracking  HRV |  | 69% | Changes to physiology and technical skills in response to stressors are correlated and indicate a relationship between psychomotor skills and personal resourcefulness (e.g., attention, stress management) |
| Jukes et al. ^5^ 2017. | Australia, Chile | ENT | Measure task demands  Stress quantification | To quantify the stress response to a simulation model, and to determine its efficacy | n= 40  Surgeons (Mixed ability - unspecified) | Open | Stress | BP  HR |  | 56% | BP and HR changes compared to baseline indicating a realistic increased stress response during this simulation |
| Wetzel et al. 2011. | Australia | Vascular | Evaluate a non-training intervention | To evaluate a novel stress management intervention for surgeons | n = 16  Trainee Surgeons | Open | Stress | HRV | OTAS | 38% | The intervention group showed reduced stress as reflected in HRV |
| Manzey et al. 2011. | Germany | ENT | Examine performance modulation | To investigate the consequences on performance, SA and skills acquisition using navigated-control assistance | n= 35  Medical Students | Open | Cognitive Load  Situation Awareness | BP  HRV  RR | NASA-TLX, Situation Awareness Global Assessment Technique ^93^ | 48% | Navigated control can reduce intraoperative risk, complications, and the physiological effort of the surgeon, but also reduces Situation Awareness |
| Feeley et al. 2022 | Ireland | Orthopaedics | Evaluate training efficacy | To evaluate the cumulative impact of supervision on technical skills and surrogate stress markers in trainees | n = 20  Trainee Surgeons | Open | Stress | Calories burned  HR |  | 67% | Surgical training pathways may derive benefit from simulation-based training in trainees. Trainees exhibit increased stress during supervised training. |
| Plazak et al. 2019. | Canada | Neurosurgery | Compare different surgical modalities | To examine cognitive load associations with utilising auditory display within image-guided neurosurgery | n = 13  Non-expert participants | Open (Neuro-  navigation) | Cognitive Load | EEG | NASA-TLX | 55% | Significant differences in Cognitive Load across modalities were detecting using EEG |
| Wetzel et al. 2010. | UK; Australia | General Surgery, Vascular Surgery | Examine performance modulation  Evaluate a non-training intervention | To investigate the effects of surgeons’ stress levels and coping strategies on surgical performance during simulated operations | n = 30  Surgeons (Mixed ability - unspecified) | Open | Stress | HRV | OTAS | 50% | Stress and coping influenced surgical performance during simulated operations |
| Wu et al. 2021. | USA | Urology | Measure task demands  Evaluate training efficacy | To measure changes in cognitive load and behaviours as they progress through robotic training programme | n = 7  Trainee Surgeons, Medical Students | Robotic | Cognitive Load  Situation Awareness | EEG  Eye-metrics: Pupillometry Eye-tracking | NASA-TLX | 79% | Changes in cognitive and behavioural states were accurately able to predict training outcomes suggesting that these metrics correlate with performance |
| Zhou et al. 2020. | USA | Urology | User testing in technological innovation | Design a framework to leverage wireless sensors to monitor surgeons' cognitive load and predict their cognitive states | n = 12  Trainee Surgeons | Robotic | Cognitive Load | EDA  EEG  EMG  HRV | NASA-TLX | 46% | The multi-sensor approach outperformed individual measurements and can correctly predict Cognitive Load 83.2% of the time |
| Shafiei et al. 2020. | USA | Not declared (Robotic Tasks) | User testing in technological innovation | To develop an objective cognitive load evaluation method using EEG data | n = 27  Trainee Surgeon (26), Fully Qualified Surgeon (1) | Robotic | Cognitive Load | EEG | NASA-TLX | 67% | Automatic detection and classification of Cognitive Load changes using EEG is feasible |
| Shafiei et al. 2021. | USA | Not declared (Robotic Tasks) | User testing in technological innovation | To develop an algorithm for objective evaluation of distraction of surgeons during robot-assisted surgery | n = 22  Medical Students | Robotic | Situation Awareness | EEG | SURG-TLX | 57% | EEG measurements detect distraction levels accurately. Distraction levels are correlated with Situational Awareness and performance |
| Wu et al. 2020. | USA | Not declared (Robotic Tasks) | User testing in technological innovation  Cognitive Load quantification | To assess the relationship between eye-tracking measures and perceived workload in robotic surgical tasks | n = 8  Trainee Surgeons | Robotic | Cognitive Load | Eye-metrics: Eye-tracking | NASA-TLX | 50% | Eye-tracking changes correlate with subjective Cognitive Load and can be used to identify task demand on Cognitive Load and provide training measures |
| Hurley et al. 2015. | Ireland | Gynaecology | Task demands | To assess the impact on physiological stress levels whilst performing surgical tasks | n = 16  Medical Students | Robotic | Stress | BP  EDA  HR  HRV |  | 57% | Stress impairs technical and non-technical skills as reflected in physiological metrics |
| Roberts et al. 2022 | USA | Urology | Analyze patient safety  Individual performance assessment | To understand the relationship between technical skills, cognitive load, and errors during simulated robotics | n = 22  Surgeons (Mixed ability - unspecified) | Robotic | Cognitive Load | Eye-metrics: Pupillometry |  | 55% | Found an optimal Cognitive Load level for surgeons during a robotic surgical exercise using Eye-metrics |
| Guru et al. 2015. | USA | Urology | User testing in technological innovation | To investigate the utility of cognitive assessment during robot-assisted surgery to define skills | n = 10  Trainee Surgeon (5), Fully Qualified Surgeon (5) | Robotic | Cognitive Load  Cognitive skills | EEG |  | 62% | Cognitive assessment of surgeons may aid in defining levels of expertise performing complex tasks |
| Singh et al. 2018. | UK | General Surgery | Measure task demands | To assess changes in neurological (fNIRS) measurements during a suturing task | n = 8  Trainee Surgeon (7), Fully Qualified Surgeon (1) | Mixture (Laparoscopic and Robotic) | Cognitive Load | fNIRs  HRV | SURG-TLX | 46% | Robotic surgery improves performance during high workload conditions and is associated with enhanced activation in regions of attention, concentration, and task engagement |
| Moore et al. 2015. | UK | Not declared (Laparoscopic and Robotic Tasks) | Compare different surgical modalities | To assess the benefits of robotic surgery compared to laparoscopy in regard to mental effort and workload | n = 32  Trainee Surgeon (8), Fully Qualified Surgeon (24) | Mixture (Robotic and Laparoscopic) | Cognitive Load | Eye-metrics: Eye-tracking  HRV | SURG-TLX | 56% | Decreased mental effort and Cognitive Load was evident when using the robotics over laparoscopy. |
| Morales et al. 2019. | Spain | General Surgery, Urology | User testing in technological innovation  Cognitive Load quantification | To assess the sensitivity of an EEG-based index, to quantify the mental workload in surgeons in real scenarios | n = 8  Fully Qualified Surgeon | Mixture (Minimally invasive) | Cognitive Load | EEG | NASA-TLX | 64% | EEG metrics can assess Cognitive Load and monitor surgeons cognitive state during training |
| Diaz-Piedra et al. 2017. | Spain | Urology | User testing in technological innovation | To assess the sensitivity of gaze-base metrics in detecting cognitive demands imposed by surgical procedures | n = 15  Surgeons (Mixed ability - unspecified) | Endourology | Cognitive Load | Eye-metrics: Eye-tracking | NASA-TLX | 56% | Gaze-based metrics can assess Cognitive Load, and act as a complementary assessment tool to quantify the learning curve |
| Anschuetz et al. 2019. | Netherlands | ENT | Compare different surgical modalities | To investigate the usability, advantages and disadvantages of 3D v 2D endoscopy in EES under standardized conditions | n = 16  Surgeons (Mixed ability - unspecified) | Endoscopic | Cognitive Load | Eye-metrics: Eye-tracking, Blinks |  | 60% | Assessment of Cognitive Load after each task revealed a lower cognitive load with increasing experience |
| Kratzke et al. 2021. | USA | Not declared (Working Memory Task) | Support provider wellbeing  Evaluate a non-training intervention | To assess the impact of neurofeedback on the cognitive load of surgery residents with burnout and depression | n = 15  Trainee Surgeons | Memory task | Cognitive Load | EEG |  | 46% | The neurofeedback intervention group showed a significant improvement in Cognitive Load via EEG |
| James et al. 2021. | UK | Not declared ("Surgical disciplines") | Stress quantification  Evaluate training efficacy | To measure physiological stress response during simulation training | n = 10  Trainee Surgeons | Surgical skills simulation | Stress | HR  RR |  | 40% | Simulation training was associated with significant and varied physiological stress responses |
| Phitayakorn et al. 2015. | USA | General Surgery | Support provider wellbeing  Team performance assessment | To compare physiological and psychological anxiety assessments in OR teams during simulated events | n= 121  Surgeons (Mixed ability - unspecified, as part of mixed profession team) | Open (Airway Emergency) | Cognitive Load  Teamwork  Situation Awareness | EDA |  | 42% | Physiological signs of Cognitive Load that do not correlate to self-reported psychological measurements |
| Hall et al. 2021. | Wales; USA | Paediatric ENT | Measure task demands | To investigate surgeons’ response to a stressful event within a simulated paediatric emergency | n = 20  Surgeons (Mixed ability - unspecified) | Undeclared - paediatric ENT Simulations | Communication | Acoustic analysis |  | 62% | Acoustic analysis of participants offers a simple, non-invasive, non-intrusive adjunct to evaluate communication and the stress response during simulation |
| Presti et al. 2021 | Italy | Not declared | User testing in technological innovation | To assess the capability of a multisensory platform to accurately measure various signals simultaneously | n = 1  Not declared | Not specified ("Surgical Tasks") | Teamwork (Cognitive Intelligence) | EDA  HR  Movement  RR |  | 40% | Physiological signals and kinematics can be used to note events occurring during surgery and be used for team performance evaluation |
| Key: **QATSSD:** Quality Assessment Tool for Diverse Designs^80^: QATSDD scores evaluates quantitative, qualitative, and mixed-method studies based on 16 criteria relating to study design, data analysis and critical appraisal. A study may be assigned a QATSDD score of between 0 and 48 if mixed-methods, and between 0 and 42 if quantitative, with higher scores indicating higher quality studies. **EDA:** Electro-Dermal Activity; **HR:** Heart Rate; **HRV**: Heart Rate Variability; **fNIRS**: Functional Near-Infrared Spectroscopy; **METs**: Metabolic Equivalents; **RR**: Respiratory Rate; **BP**: Blood Pressure; **EEG:** Electroencephalography; **EMG:** Electromyography; **NASA-TLX:** NASA Task-Load Index^79^; **OTAS**: Observational Teamwork Assessment for Surgery^136^; **SURG-TLX**: Surgical Task-Load Index^72^; **R-TLX**: Raw Task-Load index | | | | | | | | | | | |

| Supplemental Table 2: Mapping Sabermetric concepts and biomarkers against NOTSS categories. This demonstrates that physiological measurements have been used as proxies of surgical situation awareness, communication, teamwork and leadership - but metrics assessing intraoperative decision making are currently absent from the literature. | | | | |
| --- | --- | --- | --- | --- |
| NOTSS category | NOTSS Domain | Sabermetric concepts | Sabermetric biomarkers | Study examples |
| Situation Awareness | Gathering information | Eye-gaze pattern | Eye-metrics | Erridge et al. 2018^78^, He et al. 2021^77^ |
|  |  | Distractions causing changes to Cognitive Load | EDA, temperature change, Eye-metrics, HR, HRV, fNIRS | Wilson et al. 2021^94^, Pluyter et al. 2013^43^, Gao et al. 2018^95^, Gao et al. 2019^101^, Engelmann et al. 2014^88^, Kennedy-Metz et al. 2022^91^, Modi et al. 2019^97^ |
|  | Understanding information | Changes to Cognitive Load | HRV, EDA, Eye-metrics, Temperature, HR, HRV, METs, RR, BP | van Houwelingen et al. 2020^87^, Manzey et al. 2011^90^, Lechappe et al. 2020^96^, Phitayakorn et al. 2015^43^ |
|  |  | Engagement or attention | EEG, Eye-metrics, | Wu et al. 2021^91^, Kahol et al. 2011^97^ |
|  | Projecting and anticipating future state |  |  |  |
| Decision Making | Considering options |  |  |  |
|  | Selecting and communicating options |  |  |  |
|  | Implementing and reviewing decisions |  |  |  |
| Communication and Teamwork | Exchanging information | Noise levels | Acoustic Analysis | Kennedy-Metz et al. 2022^112^ |
|  | Establishing a shared understanding | Shared Cognitive Load | Eye-metrics, HR, HRV, EDA | He et al. 2021^137^, Kennedy-Metz et al. 2022^72^, Lechappe et al. 2020^78^, Kennedy-Metz et al. 2021^138^, Dias et al. 2019^7^, Phitayakorn et al. 2015^43^ |
|  |  | Shared Gaze patterns | Eye-metrics | He et al. 2021^97^ |
|  | Co-ordinating team activities | Pitch (speech) | Acoustic Analysis | Cha et al. 2022^33^ |
| Leadership | Setting and maintain standards |  |  |  |
|  | Supporting others | Mentorship and cognitive engagement with trainee task | EEG | Shafiei et al. 2018^112^, Hussein et al. 2016^137^ |
|  | Coping with pressure | Stress resilience and management | fNIRS, HRV, HR, BP | Zenati et al. 2019^46^, Modi et al. 2019^78^, Jukes et al. 2017^138^, Wetzel et al. 2011^42^ |

Key: **EDA:** Electro-Dermal Activity; **HR:** Heart Rate; **HRV**: Heart Rate Variability; **fNIRS**: Functional Near-Infrared Spectroscopy; **METs**: Metabolic Equivalents; **RR**: Respiratory Rate; **BP**: Blood Pressure

# Appendixes

Appendix A: PubMed Search Terms

Appendix B: Full list of references for included studies

Appendix C: PRISMA ScR Checklist

# Appendix A: PubMed Search Terms

(surgeon* OR "operating room" or "operating theatre") AND ("non-technical skills"or "nontechnical skills" or cognitive overload or mental overload or mental workload or cognitive load or mental load or communication skills or "situational awareness" or stressors or distractions or interruptions or teamwork or leadership or decision making or problem solving or clinical reasoning) AND (measur* or evaluat* or monitor* or assess* or digital or data or real-time or technology or continuous or sensors or physiolog* or objective)

# Appendix B: Full list of references for included papers

| Author | Full Reference |
| --- | --- |
| Anschuetz et al. 2019. | Anschuetz L, Niederhauser L, Wimmer W, et al. Comparison of 3- vs 2-Dimensional Endoscopy Using Eye Tracking and Assessment of Cognitive Load Among Surgeons Performing Endoscopic Ear Surgery. *JAMA Otolaryngol Head Neck Surg*. 2019;145:838–845. |
| Arora et al.  2010. | Arora S, Tierney T, Sevdalis N, et al. The Imperial Stress Assessment Tool (ISAT): A Feasible, Reliable and Valid Approach to Measuring Stress in the Operating Room. *World J Surg*. 2010;34:1756–1763. |
| Cha et al. 2022 | Cha JS, Athanasiadis DI, Peng Y, et al. Objective Nontechnical Skills Measurement Using Sensor-based Behavior Metrics in Surgical Teams. *Hum Factors*. 2022;187208221101292. |
| Di Stasi et al.  2016. | Di Stasi LL, Diaz-Piedra C, Rieiro H, et al. Gaze entropy reflects surgical task load. *Surg Endosc*. 2016;30:5034–5043. |
| Dias et al.  2018. | Dias RD, Osterweil LJ, Riccardi G, et al. Development of an Interactive Dashboard to Analyze Cognitive Workload of Surgical Teams During Complex Procedural Care. *IEEE International Inter-Disciplinary Conference on Cognitive Methods in Situation Awareness and Decision Support IEEE International Multi-Disciplinary Conference on Cognitive Methods in Situation Awareness and Decision Support*. 2018;2018:77–82. |
| Dias et al.  2019. | Dias RD, Zenati MA, Stevens R, et al. Physiological synchronization and entropy as measures of team cognitive load. *J Biomed Inform*. 2019;96:103250–103250. |
| Dias et al. 2022 | Dias RD, Kennedy-Metz LR, Yule SJ, et al. Assessing Team Situational Awareness in the Operating Room via Computer Vision. In: 2022 IEEE Conference on Cognitive and Computational Aspects of Situation Management (CogSIMA). 2022:94–96. |
| Diaz-Piedra et al.  2017. | Diaz-Piedra C, Sanchez-Carrion JM, Rieiro H, et al. Gaze-based Technology as a Tool for Surgical Skills Assessment and Training in Urology. *Urology*. 2017;107:26–30. |
| Ducarme et al.  2015. | Ducarme G, Bricou A, Chanelles O, et al. Stress Experienced by Obstetrics and Gynecology Residents during Planned Laparoscopy: A Prospective, Multicentric, Observational, Blinded, and Comparative Study. *Gynecol Obstet Invest*. 2015;80:148–152. |
| Duru et al. 2013. | Duru DG, Deniz Duru A, Barkana DE, et al. Assessment of surgeon’s stress level and alertness using EEG during laparoscopic simple nephrectomy. In: 2013 6th International IEEE/EMBS Conference on Neural Engineering (NER). IEEE. Epub ahead of print November 2013. DOI: 10.1109/ner.2013.6695969. |
| Engelmann et al. 2011. | Engelmann C, Schneider M, Kirschbaum C, et al. Effects of intraoperative breaks on mental and somatic operator fatigue: a randomized clinical trial. *Surg Endosc*. 2011;25:1245–1250. |
| Engelmann et al. 2014 | Engelmann CR, Neis JP, Kirschbaum C, et al. A noise-reduction program in a pediatric operation theatre is associated with surgeon’s benefits and a reduced rate of complications: a prospective controlled clinical trial. *Ann Surg*. 2014;259:1025–1033. |
| Erestam et al.  2021. | Erestam S, Bock D, Andersson AE, et al. The perceived benefit of intraoperative stress modifiers for surgeons: an experimental simulation study in volunteers. *Patient Saf Surg*. 2021;15:23–23. |
| Erridge et al. 2018. | Erridge S, Ashraf H, Purkayastha S, et al. Comparison of gaze behaviour of trainee and experienced surgeons during laparoscopic gastric bypass. *Br J Surg*. 2018;105:287–294. |
| Feeley et al. 2022 | Feeley AA, Feeley IH, McManus R, et al. Evaluating the Impact of Supervision on Surgical Trainees Stress Response During Simulated Surgical Procedures; A Crossover Randomized Trial. *J Surg Educ*.. Epub ahead of print July 30, 2022. DOI: 10.1016/j.jsurg.2022.07.006. |
| Gao et al.  2018. | Gao J, Liu S, Feng Q, et al. Quantitative Evaluations of the Effects of Noise on Mental Workloads Based on Pupil Dilation during Laparoscopic Surgery. *Am Surg*. 2018;84:1951–1956. |
| Gao et al.  2019. | Gao J, Liu S, Feng Q, et al. Subjective and Objective Quantification of the Effect of Distraction on Physician’s Workload and Performance During Simulated Laparoscopic Surgery. *Med Sci Monit*. 2019;25:3127–3132. |
| Grantcharov et al.  2019. | Grantcharov PD, Boillat T, Elkabany S, et al. Acute mental stress and surgical performance. *BJS open*. 2019;3:119–125. |
| Gunawardena et al. 2019. | Gunawardena N, Matscheko M, Anzengruber B, et al. Assessing surgeons’ skill level in laparoscopic cholecystectomy using eye metrics. Association for Computing Machinery; 2019:Article 30. |
| Guru et al.  2015. | Guru KA, Shafiei SB, Khan A, et al. Understanding Cognitive Performance During Robot-Assisted Surgery. *Urology*. 2015;86:751–757. |
| Guru et al.  2015. | Guru KA, Esfahani ET, Raza SJ, et al. Cognitive skills assessment during robot-assisted surgery: separating the wheat from the chaff. *BJU Int*. 2015;115:166–174. |
| Guzmán-García et al. 2022 | Guzmán-García C, Sánchez-González P, Margallo JAS, et al. Correlating Personal Resourcefulness and Psychomotor Skills: An Analysis of Stress, Visual Attention and Technical Metrics. *Sensors* .;22. Epub ahead of print January 22, 2022. DOI: 10.3390/s22030837. |
| Hall et al.  2021. | Hall A, Kawai K, Graber K, et al. Acoustic analysis of surgeons’ voices to assess change in the stress response during surgical in situ simulation. *BMJ Simulation & Technology Enhanced Learning*.;7 Available from: https://access.portico.org/Portico/show?content=E-Journal%20Content&cs=ISSN_20566697_1218&auId=ark%3A%2F27927%2Fphzpr205jnv&auViewType1=PDF&auViewType2=PDF. 2021. |
| He et al.  2021. | He W, Jiang X, Zheng B. Synchronization of Pupil Dilations Correlates With Team Performance in a Simulated Laparoscopic Team Coordination Task. *Simul Healthc*. 2021;16:e206–e213. |
| Hurley et al.^6^ 2015. | Hurley AM, Kennedy PJ, O’Connor L, et al. SOS save our surgeons: Stress levels reduced by robotic surgery. *Gynecol Surg*. 2015;12:197–206. |
| Hussein et al.  2016. | Hussein AA, Shafiei SB, Sharif M, et al. Technical mentorship during robot‐assisted surgery: a cognitive analysis. *BJU Int*. 2016;118:429–436. |
| James et al.  2021. | James OP, Robinson DBT, Hopkins L, et al. Biosensors, biomarkers and biometrics: A bootcamp perspective. *BMJ Simul Technol Enhanc Learn*. 2020;bmjstel–2020–000631. |
| Jevsevar et al.  2021. | Jevsevar DS, Molloy IB, Gitajn IL, et al. Orthopaedic Surgeon Physiological Indicators of Strain as Measured by a Wearable Fitness Device. *J Am Acad Orthop Surg*. 2021;29:e1378–e1386. |
| Ji et al. 2022 | Ji Y, Kong Z, Deng Y, et al. The role of eye tracker in teaching video-assisted thoracoscopic surgery: the differences in visual strategies between novice and expert surgeons in thoracoscopic surgery. *Ann Transl Med*. 2022;10:592. |
| Jiang et al.  2013. | Jiang X, Zheng B, Tien G, et al. Pupil response to precision in surgical task execution. *Stud Health Technol Inform*. 2013;184:210–214. |
| Jukes et al.  2017. | Jukes AK, Mascarenhas A, Murphy J, et al. Stress response and communication in surgeons undergoing training in endoscopic management of major vessel hemorrhage: a mixed methods study. *Int Forum Allergy Rhinol*. 2017;7:576–583. |
| Kahol et al. 2011. | Kahol K, Smith M, Brandenberger J, et al. Impact of fatigue on neurophysiologic measures of surgical residents. *J Am Coll Surg*. 2011;213:29–34; discussion 34–6. |
| Kelkar et al. 2021 | Kelkar A, Kelkar J, Chougule Y, et al. Cognitive workload, complications and visual outcomes of phacoemulsification cataract surgery: Three-dimensional versus conventional microscope. *Eur J Ophthalmol*. 2022;32:2935–2941. |
| Kennedy-Metz et al.  2021. | Kennedy-Metz LR, Dias RD, Stevens RH, et al. Analysis of Mirrored Psychophysiological Change of Cardiac Surgery Team Members During Open Surgery. *J Surg Educ*. 2021;78:622–629. |
| Kennedy-Metz et al. 2020. | Kennedy-Metz LR, Dias RD, Srey R, et al. Sensors for Continuous Monitoring of Surgeon’s Cognitive Workload in the Cardiac Operating Room. *Sensors* . 2020;20:6616. |
| Kennedy-Metz et al. 2021. | Kennedy-Metz L, Dias R, Zenati M. The Cognitive Relevance of a Formal Pre-incision Time-out in Surgery. In: Proceedings of the 32nd European Conference on Cognitive Ergonomics (ECCE ’21). New York, NY: Association for Computing Machinery; 2021:1–5. |
| Kennedy-Metz et al. 2022 | Kennedy-Metz LR, Arshanskiy M, Keller S, et al. Association Between Operating Room Noise and Team Cognitive Workload in Cardiac Surgery. In: 2022 IEEE Conference on Cognitive and Computational Aspects of Situation Management (CogSIMA). 2022:89–93. |
| Kratzke et al.  2021. | Kratzke IM, Campbell A, Yefimov MN, et al. Pilot Study Using Neurofeedback as a Tool to Reduce Surgical Resident Burnout. *J Am Coll Surg*. 2021;232:74–80. |
| Lechappe et al.  2020. | Lechappe A, Chollet M, Rigaud J, et al. Assessment of Situation Awareness during Robotic Surgery using Multimodal Data. Association for Computing Machinery; 2020:412–416. |
| Louridas et al. 2015. | Louridas M, Bonrath EM, Sinclair DA, et al. Randomized clinical trial to evaluate mental practice in enhancing advanced laparoscopic surgical performance. *Br J Surg*. 2015;102:37–44. |
| Malmon et al. 2021 | Maimon NB, Bez M, Drobot D, et al. Continuous monitoring of mental load during virtual simulator training for laparoscopic surgery reflects laparoscopic dexterity. A comparative study using a novel wireless device. *Front Neurosci*. 2022;15:1716. |
| Manzey et al.  2011. | Manzey D, Luz M, Mueller S, et al. Automation in surgery: the impact of navigated-control assistance on performance, workload, situation awareness, and acquisition of surgical skills. *Hum Factors*. 2011;53:584–599. |
| Modi et al.  2019. | Modi HN, Singh H, Fiorentino F, et al. Association of Residents’ Neural Signatures With Stress Resilience During Surgery. *JAMA Surg*. 2019;154:e192552. |
| Modi et al.  2020. | Modi HN, Singh H, Darzi A, et al. Multitasking and Time Pressure in the Operating Room: Impact on Surgeons’ Brain Function. *Ann Surg*. 2020;272:648. |
| Modi et al. ^28^ 2018. | Modi HN, Singh H, Orihuela-Espina F, et al. Temporal Stress in the Operating Room: Brain Engagement Promotes “Coping” and Disengagement Prompts “Choking.” *Ann Surg*. 2018;267:683–691. |
| Moore et al.^3^ 2015. | Moore LJ, Wilson MR, McGrath JS, et al. Surgeons’ display reduced mental effort and workload while performing robotically assisted surgical tasks, when compared to conventional laparoscopy. *Surg Endosc*. 2015;29:2553–2560. |
| Morales et al.^4^ 2019. | Morales JM, Ruiz-Rabelo JF, Diaz-Piedra C, et al. Detecting Mental Workload in Surgical Teams Using a Wearable Single-Channel Electroencephalographic Device. *J Surg Educ*. 2019;76:1107–1115. |
| Phitayakorn et al.  2015. | Phitayakorn R, Minehart RD, Hemingway MW, et al. Relationship between physiologic and psychological measures of autonomic activation in operating room teams during a simulated airway emergency. *Am J Surg*. 2015;209:86–92. |
| Pimentel et al.  2019. | Pimentel G, Rodrigues S, Silva PA, et al. A wearable approach for intraoperative physiological stress monitoring of multiple cooperative surgeons. *Int J Med Inform*. 2019;129:60–68. |
| Plazak et al.  2019. | Plazak J, DiGiovanni DA, Collins DL, et al. Cognitive load associations when utilizing auditory display within image-guided neurosurgery. *Int J Comput Assist Radiol Surg*. 2019;14:1431–1438. |
| Pluyter et al.  2013. | Pluyter JR, Rutkowski A, Jakimowicz J. Immersive training: breaking the bubble and measuring the heat. *Surg Endosc*. 2013;28:1545–1554. |
| Poolton et al.  2011. | Poolton JM, Wilson MR, Malhotra N, et al. A comparison of evaluation, time pressure, and multitasking as stressors of psychomotor operative performance. *Surgery*. 2011;149:776–782. |
| Presti et al. 2021 | Presti DL, Gravina R, Massaroni C, et al. A Multisensory Platform for Maximizing Collective Intelligence in the Operating Room. In: 2021 IEEE/ACM Conference on Connected Health: Applications, Systems and Engineering Technologies (CHASE). 2021:174–178. |
| Rieger et al.  2015. | Rieger A, Fenger S, Neubert S, et al. Psychophysical workload in the operating room: primary surgeon versus assistant. *Surg Endosc*. 2015;29:1990–1998. |
| Roberts et al. 2022 | Roberts SI, Cen SY, Nguyen JH, et al. The Relationship Between Technical Skills, Cognitive Workload, and Errors During Robotic Surgical Exercises. *J Endourol*. 2022;36:712–720. |
| Shafiei et al.  2020. | Shafiei SB, Elsayed AS, Hussein AA, et al. Evaluating the Mental Workload During Robot-Assisted Surgery Utilizing Network Flexibility of Human Brain. *IEEE Access*. undefined 2020;8:204012–204019. |
| Shafiei et al. 2018. | Shafiei SB, Hussein AA, Muldoon SF, et al. Functional Brain States Measure Mentor-Trainee Trust during Robot-Assisted Surgery. *Sci Rep*. 2018;8:3667–3612. |
| Shafiei et al.^0^ 2021. | Shafiei SB, Iqbal U, Hussein AA, et al. Utilizing deep neural networks and electroencephalogram for objective evaluation of surgeon’s distraction during robot-assisted surgery. *Brain Res*. 2021;1769:147607. |
| Shafiei, et al. 2021. | Shafiei SB, Jing Z, Attwood K, et al. Association between Functional Brain Network Metrics and Surgeon Performance and Distraction in the Operating Room. *Brain Sci*.;11. Epub ahead of print April 8, 2021. DOI: 10.3390/brainsci11040468. |
| Singh et al.^8^ 2018. | Singh H, Modi HN, Ranjan S, et al. Robotic Surgery Improves Technical Performance and Enhances Prefrontal Activation During High Temporal Demand. *Ann Biomed Eng*. 2018;46:1621–1636. |
| Stevens et al.  2019. | Stevens R, Galloway T, Willemsen-Dunlap A. Advancing Our Understandings of Healthcare Team Dynamics From the Simulation Room to the Operating Room: A Neurodynamic Perspective. *Front Psychol*. 2019;10:1660. |
| Suarez-Revelo et al.  2019. | Suarez-Revelo JX, Ochoa-Gomez JF, Hernandez-Valdivieso AM. Neurophysiological changes associated with training in laparoscopic surgery using EEG: a pilot study. *Conf Proc IEEE Eng Med Biol Soc*. 2019;2019:4572–4575. |
| Theodoraki et al. 2015. | Theodoraki MN, Ledderose GJ, Becker S, et al. Mental distress and effort to engage an image-guided navigation system in the surgical training of endoscopic sinus surgery: a prospective, randomised clinical trial. *Eur Arch Otorhinolaryngol*. 2015;272:905–913. |
| Tien et al.  2015. | Tien T, Pucher PH, Sodergren MH, et al. Differences in gaze behaviour of expert and junior surgeons performing open inguinal hernia repair. *Surg Endosc*. 2015;29:405–413. |
| Uslu et al. 2018. | Uslu S, Atici, Göğüs Y, et al. Stress In the Operating Room: Emergency and Elective Surgeries. *Anestezi Dergisi*. 2018;26:127–131. |
| van Houwelingen et al.  2020. | van Houwelingen BCG, Rutkowski A-F, Ganni S, et al. Effects of surgical flow disruptions on surgeons’ resources: a pilot study. *Surg Endosc*. 2020;34:4525–4535. |
| Wetzel et al.  2010. | Wetzel CM, Black SA, Hanna GB, et al. The Effects of Stress and Coping on Surgical Performance During Simulations. *Ann Surg*. 2010;251:171–176. |
| Wetzel et al. 2011. | Wetzel CM, George A, Hanna GB, et al. Stress Management Training for Surgeons—A Randomized, Controlled, Intervention Study. *Ann Surg*. 2011;253:488–494. |
| Wilson et al. 2021. | Wilson C, Chahine S, Cristancho S, et al. Unusual suspects: Real-time physiological evaluation of stressors during laparoscopic donor nephrectomy. *Can Urol Assoc J*. 2021;15:E205–E209. |
| Wu et al.  2020. | Wu C, Cha J, Sulek J, et al. Eye-Tracking Metrics Predict Perceived Workload in Robotic Surgical Skills Training. *Hum Factors*. 2020;62:1365–1386. |
| Wu et al.  2021. | Wu C, Cha J, Sulek J, et al. Sensor-based indicators of performance changes between sessions during robotic surgery training. *Appl Ergon*. 2021;90:103251. |
| Yu  et al.  2021. | Yu P, Pan J, Wang Z, et al. Cognitive Load/flow and Performance in Virtual Reality Simulation Training of Laparoscopic Surgery. In: 2021 IEEE Conference on Virtual Reality and 3D User Interfaces Abstracts and Workshops (VRW). 2021:466–467. |
| Yu et al. 2022 | Yu P, Pan J, Wang Z, et al. Quantitative influence and performance analysis of virtual reality laparoscopic surgical training system. *BMC Med Educ*. 2022;22:92. |
| Zakeri et al.  2020. | Zakeri Z, Mansfield N, Sunderland C, et al. Physiological correlates of cognitive load in laparoscopic surgery. *Sci Rep*. 2020;10:12927–12927. |
| Zenati et al. 2019. | Zenati MA, Leissner KB, Zorca S, et al. First Reported Use of Team Cognitive Workload for Root Cause Analysis in Cardiac Surgery. *Semin Thorac Cardiovasc Surg*. 2019;31:394–396. |
| Zhang et al.  2017. | Zhang J-Y, Liu S-L, Feng Q-M, et al. Correlative Evaluation of Mental and Physical Workload of Laparoscopic Surgeons Based on Surface Electromyography and Eye-tracking Signals. *Sci Rep*. 2017;7:11095–11097. |
| Zhang et al. 2021. | Zhang J-Y, Shen Z-H, Wang B-P, et al. Influence of 3D laparoscopic surgery on surgeon’s visual pattern and mental workload. *J Med Eng Technol*. 2021;45:375–379. |
| Zhang et al.^9^ 2018. | Zhang J, Liu S, Feng Q, et al. Ergonomic Assessment of the Mental Workload Confronted by Surgeons during Laparoscopic Surgery. *Am Surg*. 2018;84:1538–1543. |
| Zheng et al.  2012. | Zheng B, Jiang X, Tien G, et al. Workload assessment of surgeons: correlation between NASA TLX and blinks. *Surg Endosc*. 2012;26:2746–2750. |
| Zheng et al. 2011 | Zheng B, Tien G, Atkins SM, et al. Surgeon’s vigilance in the operating room. *Am J Surg*. 2011;201:673–677. |
| Zhou et al.2020. | Zhou T, Cha JS, Gonzalez G, et al. Multimodal Physiological Signals for Workload Prediction in Robot-assisted Surgery. *ACM transactions on human-robotic interaction*. 2020;9:Article 12. |

# Appendix C: PRISMA-ScR checklist

**Preferred Reporting Items for Systematic reviews and Meta-Analyses extension for Scoping Reviews (PRISMA-ScR) Checklist**^1^

| **SECTION** | **ITEM** | **PRISMA-ScR CHECKLIST ITEM** | **REPORTED ON PAGE #** |
| --- | --- | --- | --- |
| **TITLE** | | | |
| Title | 1 | Identify the report as a scoping review. | 1 |
| **ABSTRACT** | | | |
| Structured summary | 2 | Provide a structured summary that includes (as applicable): background, objectives, eligibility criteria, sources of evidence, charting methods, results, and conclusions that relate to the review questions and objectives. | 3 |
| **INTRODUCTION** | | | |
| Rationale | 3 | Describe the rationale for the review in the context of what is already known. Explain why the review questions/objectives lend themselves to a scoping review approach. | 5 |
| Objectives | 4 | Provide an explicit statement of the questions and objectives being addressed with reference to their key elements (e.g., population or participants, concepts, and context) or other relevant key elements used to conceptualize the review questions and/or objectives. | 6 |
| **METHODS** | | | |
| Protocol and registration | 5 | Indicate whether a review protocol exists; state if and where it can be accessed (e.g., a Web address); and if available, provide registration information, including the registration number. | 6 |
| Eligibility criteria | 6 | Specify characteristics of the sources of evidence used as eligibility criteria (e.g., years considered, language, and publication status), and provide a rationale. | 6-7 |
| Information sources* | 7 | Describe all information sources in the search (e.g., databases with dates of coverage and contact with authors to identify additional sources), as well as the date the most recent search was executed. | 6 |
| Search | 8 | Present the full electronic search strategy for at least 1 database, including any limits used, such that it could be repeated. | Supplemental material |
| Selection of sources of evidence† | 9 | State the process for selecting sources of evidence (i.e., screening and eligibility) included in the scoping review. | 6-7 |
| Data charting process‡ | 10 | Describe the methods of charting data from the included sources of evidence (e.g., calibrated forms or forms that have been tested by the team before their use, and whether data charting was done independently or in duplicate) and any processes for obtaining and confirming data from investigators. | 7 |
| Data items | 11 | List and define all variables for which data were sought and any assumptions and simplifications made. | Table 1 Page 20 |
| Critical appraisal of individual sources of evidence§ | 12 | If done, provide a rationale for conducting a critical appraisal of included sources of evidence; describe the methods used and how this information was used in any data synthesis (if appropriate). | 7 |
| Synthesis of results | 13 | Describe the methods of handling and summarizing the data that were charted. | 7 |
| **RESULTS** | | | |
| Selection of sources of evidence | 14 | Give numbers of sources of evidence screened, assessed for eligibility, and included in the review, with reasons for exclusions at each stage, ideally using a flow diagram. | 7,Figure 1 page 35 |
| Characteristics of sources of evidence | 15 | For each source of evidence, present characteristics for which data were charted and provide the citations. | 8 |
| Critical appraisal within sources of evidence | 16 | If done, present data on critical appraisal of included sources of evidence (see item 12). | 11 |
| Results of individual sources of evidence | 17 | For each included source of evidence, present the relevant data that were charted that relate to the review questions and objectives. | Table 1 page 20 |
| Synthesis of results | 18 | Summarize and/or present the charting results as they relate to the review questions and objectives. | Table 1-3; figures 1-3 pages 20-37 |
| **DISCUSSION** | | | |
| Summary of evidence | 19 | Summarize the main results (including an overview of concepts, themes, and types of evidence available), link to the review questions and objectives, and consider the relevance to key groups. | 11 |
| Limitations | 20 | Discuss the limitations of the scoping review process. | 13 |
| Conclusions | 21 | Provide a general interpretation of the results with respect to the review questions and objectives, as well as potential implications and/or next steps. | 17 |
| **FUNDING** | | | |
| Funding | 22 | Describe sources of funding for the included sources of evidence, as well as sources of funding for the scoping review. Describe the role of the funders of the scoping review. | 1 |

JBI = Joanna Briggs Institute; PRISMA-ScR = Preferred Reporting Items for Systematic reviews and Meta-Analyses extension for Scoping Reviews.

* Where *sources of evidence* (see second footnote) are compiled from, such as bibliographic databases, social media platforms, and Web sites.

† A more inclusive/heterogeneous term used to account for the different types of evidence or data sources (e.g., quantitative and/or qualitative research, expert opinion, and policy documents) that may be eligible in a scoping review as opposed to only studies. This is not to be confused with *information sources* (see first footnote).

‡ The frameworks by Arksey and O’Malley and Levac and colleagues and the JBI guidance refer to the process of data extraction in a scoping review as data charting*.*

§ The process of systematically examining research evidence to assess its validity, results, and relevance before using it to inform a decision. This term is used for items 12 and 19 instead of "risk of bias" (which is more applicable to systematic reviews of interventions) to include and acknowledge the various sources of evidence that may be used in a scoping review (e.g., quantitative and/or qualitative research, expert opinion, and policy document).

Reference

1. Tricco AC, Lillie E, Zarin W, et al. PRISMA Extension for Scoping Reviews (PRISMA-ScR): Checklist and Explanation. *Ann Intern Med*. 2018;169(7):467-473.
